# Accuracy and interpretability of smartwatch electrocardiogram for early detection of atrial fibrillation: A systematic review and meta‐analysis

**DOI:** 10.1002/joa3.70087

**Published:** 2025-05-22

**Authors:** Muhammad Iqhrammullah, Asnawi Abdullah, Hermansyah, Fahmi Ichwansyah, Hafnidar A. Rani, Meulu Alina, Artha M. T. Simanjuntak, Derren D. C. H. Rampengan, Seba Talat Al‐Gunaid, Naufal Gusti, Arditya Damarkusuma, Edza Aria Wikurendra

**Affiliations:** ^1^ Postgraduate Program of Public Health Universitas Muhammadiyah Aceh Banda Aceh Indonesia; ^2^ Faculty of Public Health Universitas Muhammadiyah Aceh Banda Aceh Indonesia; ^3^ Department of Applied Nursing Program Poltekkes Kemenkes Aceh Banda Aceh Indonesia; ^4^ Health Polytechnic of Aceh Ministry of Health‐Indonesia Banda Aceh Indonesia; ^5^ Department of Civil Engineering Universitas Muhammadiyah Aceh Banda Aceh Indonesia; ^6^ Faculty of Medicine Universitas Syiah Kuala Banda Aceh Indonesia; ^7^ Faculty of Medicine Universitas Gadjah Mada Yogyakarta Indonesia; ^8^ Faculty of Medicine Universitas Sam Ratulangi Manado Indonesia; ^9^ Department of Cardiology and Vascular Medicine, Faculty of Medicine, Public Health, and Nursing Universitas Gadjah Mada Yogyakarta Indonesia; ^10^ Department of Public Health, Faculty of Health Universitas Nahdlatul Ulama Surabaya Surabaya Indonesia; ^11^ Department of Health Science, Faculty of Humanities and Health Science Curtin University Miri Malaysia

**Keywords:** arrhythmia, atrial fibrillation, ECG, electrocardiogram, smartwatch

## Abstract

**Background:**

The prevalence of atrial fibrillation (AFib) continues to increase globally, posing a significant risk for serious cardiovascular complications, such as ischemic stroke and thromboembolism. Smartwatch single‐lead electrocardiogram (ECG) can be a practical and accurate early detection tool for AFib.

**Objective:**

The aim of this study was to fill the research gap in evaluating the accuracy and interpretability of smartwatch ECG for early AFib detection.

**Methods:**

Data derived from indexed literature in the Scopus, Scilit, PubMed, Google Scholar, Web of Science, IEEE, and Cochrane Library databases (as of June 1, 2024) were systematically screened and extracted. The quantitative synthesis was performed using a two‐level mixed‐effects logistic regression model, as well as a proportional analysis with Freeman‐Tukey double transformation on a restricted maximum‐likelihood model.

**Results:**

The sensitivity and specificity of smartwatch ECG in algorithmic readings were 86% and 94%, respectively. In manual readings, the sensitivity and specificity reached 96% and 95%, respectively. In a brand‐specific subgroup analysis, the algorithmic reading reached a summary area under the curve (sAUC) of 96%, while another brand achieved the highest sAUC of 98% in manual reading. The level of manual interpretability was relatively high with Cohen's Kappa of 0.83, but 3% of ECG results were difficult to read manually.

**Conclusion:**

This study shows that smartwatch ECG is able to detect AFib with high accuracy, especially through manual reading by trained medical personnel.

**PROSPERO Registration:**

CRD42024548537 (May 29, 2024).

## Graphical abstract

The smartwatch ECG algorithm achieved 86% sensitivity and 94% specificity with an sAUC of 96%, while manual readings by trained personnel reached 96% sensitivity, 95% specificity, and an sAUC of 98% in a brand‐specific subgroup analysis. These findings demonstrate that the smartwatch ECG detects AFib, although 3% of results are unreadable.

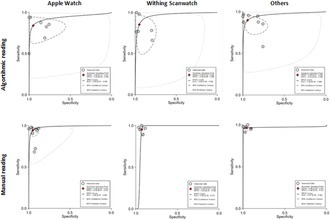

## 1 INTRODUCTION

In 2022, a global burden of cardiovascular disease study revealed that the Age‐Standardized Mortality Rate (ASMR) for cardiovascular diseases reached 73.6 per 100,000 in high‐income Asia Pacific countries.^1^ Among various cardiovascular diseases, atrial fibrillation (AFib) and flutter have a global prevalence of 637.5 per 100,000 with a mortality rate of 4.5%.^1^ The global prevalence of atrial fibrillation is projected to increase by over 60% by the year 2050 compared to the 2017 estimates.^2^

While 12‐lead electrocardiograms (ECGs) remain the gold standard for diagnosing AFib because of their comprehensive signal coverage,^3^ their use is typically limited to clinical settings. Acquiring a 12‐lead ECG requires proper electrode placement on specific anatomical landmarks, which must be done by trained professionals. In a recent usability study, clinicians experienced in ECG acquisition took an average of 3.1 minutes to complete a 12‐lead ECG using a handheld device.^4^ In contrast, smartwatch‐based single‐lead ECGs can be recorded within seconds by users themselves, without requiring clinical expertise or electrode placement.^5^, ^6^, ^7^ This ease of use, coupled with growing accessibility, makes smartwatch ECGs a promising tool for widespread AFib screening and early detection, especially in community or remote settings.

As compared to a sole photoplethysmography, single‐lead ECG in smartwatch is considered more informative because it can be clinically validated by trained medical personnel.^7^, ^8^ The ECG generated on the smartwatch can be interpreted by cardiologists with an accuracy level that may reach 100%,^8^ but the ability of the interpreters may vary depending on their experience and knowledge.^6^, ^9^ Several previous meta‐analyses have also concluded the accuracy of smartwatch ECG.^10^, ^11^ To the best of our knowledge, there was no meta‐analysis that has assessed the interpretability of smartwatch ECG in detecting AFib. Previous studies are also lacking comparative analysis on different smartwatch brands.^12^ Therefore, this study aims to compare the diagnostic capabilities of smartwatch‐based ECG, automatically by algorithms and manually by trained medical personnel, as well as to assess the interpretability.

## 2 METHODS

### 2.1 Study design and protocol registration

The study used systematic review followed by meta‐analysis to calculate the pooled estimate of sensitivity, specificity, and summary of area under the curve (sAUC) as indicators for diagnostic accuracy. Secondly, the study sought to estimate the interpretability of the smartwatch ECG by performing proportion meta‐analysis on uncertain results, nonreadable ECG, and inter‐rater kappa. The reporting of the results followed the Preferred Reporting Items for Systematic Reviews and Meta‐Analysis protocol (Checklist S1 and Checklist S2). The protocol had been registered on PROSPERO with registration number: CRD42024548537 as of May 29, 2024.

### 2.2 Eligibility criteria

Studies were included if they met the following criteria: (1) Diagnostic studies, observational studies, and randomized controlled trials (RCTs); (2) Study population consists of AFib patients. AFib was diagnosed through 12‐lead electrocardiogram readings by trained medical personnel; (3) The diagnostic tool used was a smartwatch‐based electrocardiogram with 1 lead. The study must report the ECG reading by algorithms or  trained medical personnel; (4) The results of the algorithmic and manual readings were expressed as diagnostic values. Literature that did not report diagnostic values based on comparison with a 12‐lead ECG was excluded.

### 2.3 Search method

Literature search was conducted on Scopus, Scilit, PubMed, Google Scholar, Web of Science, IEEE, and Cochrane Library as of June 1, 2024. The search included keywords such as “smartwatch,” “electrocardiogram,” and “atrial fibrillation,” which were expanded using synonyms or other equivalent terms. The keywords were then combined using the Boolean operators “AND” and “OR.” MeSH (Medical Subject Headings) terms were used in PubMed and PMC, while truncated keywords using (*) were applied in Scopus. The combination of keywords is presented in Table S1. No minimum publication year or language restrictions were applied for the literature search. Articles reported in languages other than English and Indonesian Language were excluded.

### 2.4 Screening and selection

After downloading, citation data were imported into Rayyan.ai for screening and selection. The software algorithmically identified and removed duplicate records using its built‐in duplication detection algorithm. Three independent review authors (M.I., A.M.T.S., and M.A.) then screened titles and abstracts for relevance to the research question. Full‐text assessments were conducted based on predefined inclusion and exclusion criteria. Any discrepancies in the screening and selection process were resolved through consensus.

### 2.5 Data extraction

Using a pre‐designed extraction table, data related to the characteristics of the study subjects were extracted. The data included sample size, average age, female‐to‐male ratio, average body mass index (BMI), patient conditions or settings, smartwatch brand, position of smartwatch wear, and number of assessors. Data collection was performed independently by two review authors (M.I. and A.M.T.S.). Any inconsistencies in the extracted data were resolved by re‐examining the literature and engaging in discussions until consensus was reached.

### 2.6 Quality assessment

To determine the quality of the included studies, we used Quality Assessment of Diagnostic Accuracy Studies (QUADAS)‐2.^13^ This assessment tool consists of four domains: “patient selection,” “index test,” “reference standard,” and “flow and timing.” The results were presented in a “traffic light” diagram, which indicates the quality level as “low risk,” “uncertain,” or “high risk” for each item. The visualization of the “traffic light” diagram was carried out on Review Manager 5.4.

### 2.7 Diagnostic meta‐analysis

Diagnostic meta‐analysis was performed using the “midas” package in STATA version 17. The diagnostic values of FP, FN, TN, and TP were analyzed using Spearman's correlation to determine the threshold effect based on the relationship between sensitivity and specificity. A data group is considered to have a threshold effect if the Spearman's correlation yields a *p*‐value <0.05. The specificity and sensitivity of the pooled analysis were conducted using a two‐level mixed‐effect logistic regression model. Subsequently, a summary ROC (sROC) curve was constructed to determine the summary area under the curve (sAUC). The smarwatch was considered to have high and very high accuracy if the sAUC value reaches >80%–90% and 90%–100%, respectively.^14^

### 2.8 Proportion meta‐analysis

Meta‐analysis was performed using RStudio 2024.04.2 Build 764 with the “meta” package. Similar to the previous analysis, data heterogeneity was determined based on an *I* ^2^ value >50% and *p*‐Het <0.1. The restricted maximum‐likelihood model and Freeman‐Tukey double arcsine transformation (FTT) were used to obtain the overall proportion. The estimated values were then multiplied by 100% to calculate the relative proportions. For the kappa (*κ*) coefficient, transformation using Fisher's Z and variance calculation were performed prior to the meta‐analysis. Both pooled proportion estimates and *κ* coefficient were then transformed back to their original values. Publication bias was assessed using Egger's and Begg's funnel plot correlation tests. Moderator effect analysis was conducted by examining the Z‐value and *p*‐value (significant if *p* < 0.05). This procedure was adopted from previous studies.^15^

### 2.9 Outlier identification and subgroup analysis

Outlier identification was performed through Cook's distance analysis, determined based on the formula 4/*n* (where *n* is the number of studies in the pooled analysis). Subgroup analyses were conducted based on study year, study location, age, BMI, female‐to‐male ratio, smartwatch brand, number of assessors, smartwatch wearing location, and number of assessors.

## 3 RESULTS

### 3.1 Literature selection

A literature search across eight different databases yielded 5425 records, of which 2447 were duplicates and were subsequently removed. Similarly, the search of clinical trial registries identified 65 trials, but their results were not reported, leading to their exclusion from the screening process. In the next stage, 2913 records were screened for relevance based on title and abstract, leaving 96 studies for full‐text assessment. A total of 83 studies were excluded for various reasons, which are detailed in Table S2. A manual search was then conducted by screening the reference lists of eligible studies and utilizing Connected Papers for additional sources. Among the 37 studies deemed relevant, all were accessible in full text. Screening based on inclusion and exclusion criteria determined that 33 of these 37 studies did not meet the eligibility criteria for inclusion in the review, with full details available in Table S3.

**FIGURE 1:**
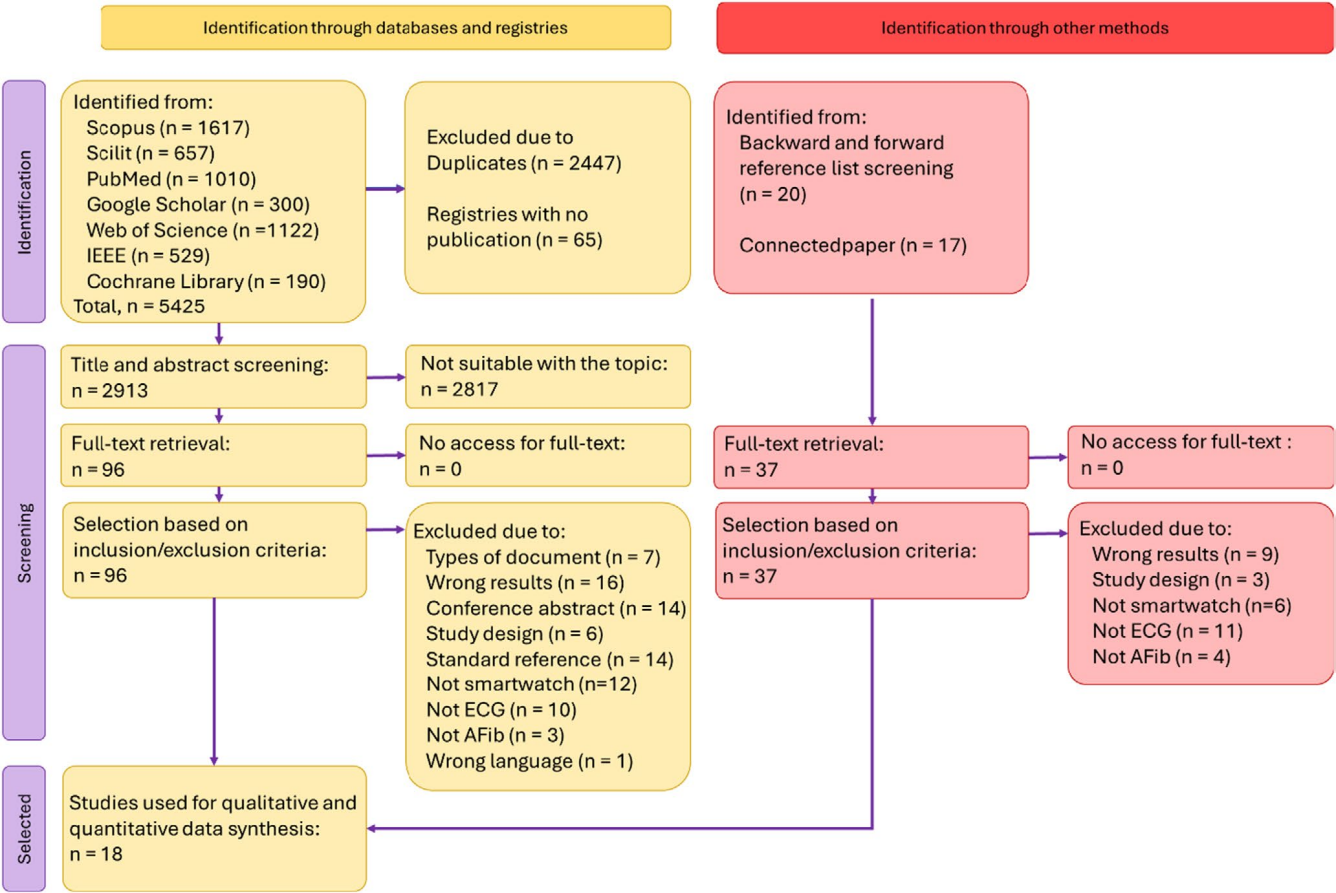
PRISMA Flow Diagram for Literature Searching and Selection Process.

Both keyword‐based and manual searches identified several articles that nearly met the eligibility criteria but were ultimately excluded after discussion. Two studies were excluded because they did not report atrial fibrillation (AFib) cases separately from atrial flutter cases.^16^, ^17^ Another study utilized the Apple Watch but relied solely on the photoplethysmography (PPG) sensor without performing an electrocardiogram (ECG) analysis.^18^ Several studies were excluded because they conducted qualitative observational research without assessing diagnostic accuracy in AFib detection.^19^, ^20^, ^21^ Others provided only theoretical reviews on smartwatch use.^22^, ^23^ The screening and selection process identified 18 studies that met the criteria for inclusion in the qualitative and quantitative synthesis.^5^, ^6^, ^7^, ^8^, ^9^, ^24^, ^25^, ^26^, ^27^, ^28^, ^29^, ^30^, ^31^, ^32^, ^33^, ^34^, ^35^, ^36^ The summary of the literature selection process is presented in Figure 1.

### 3.2 Characteristics of the included studies

The characteristics of the studies and research subjects have been summarized and presented in Table 1. Among 18 studies included in this review, only six were cohort studies,^7^, ^25^, ^31^, ^32^, ^33^, ^36^ while the remaining studies were cross‐sectional in design.^5^, ^6^, ^8^, ^9^, ^24^, ^26^, ^27^, ^28^, ^29^, ^30^, ^34^, ^35^ These studies were conducted in various countries, including France (*n* = 5), the Netherlands (*n* = 3), Switzerland (*n* = 2), Australia (*n* = 2), China (*n* = 2), Canada (*n* = 1), Portugal (*n* = 1), Norway (*n* = 1), and Turkey (*n* = 1). In all studies, the number of patients with atrial fibrillation (AFib) was smaller than the control group, except for one study.^9^ The proportion of male patients was higher than that of female patients across all studies, with the mean age ranging from 40 ± 16.6 years^33^ to 76 ± 7 years.^28^ Among the studies that reported body mass index (BMI), the average BMI values fell between >25 kg/m^2^ and <30 kg/m^2^.^25^, ^28^, ^29^, ^30^, ^37^ Some studies recruited both hospitalized and outpatient participants, including individuals with or without cardiac complaints or a history of heart disease.

**TABLE 1:** Characteristics of studies reporting the diagnostic performance of smartwatch ECG.

Author, year	Study design	Country	Patient, *n*		Baseline characteristics			Patient's status/condition
			AFib	Non‐AFib	Gender, female:Male	Age, mean ± SD	BMI, mean ± SD	
Ploux et al., 2022^24^	Cross‐sectional	France	49	211	109/151	66 ± 6	NR	Inpatient/outpatients
Abu‐Alrub et al., 2022^6^	Cross‐sectional	Canada	100	100	78/112	62 ± 7	NR	Post ablation
Mannhart et al., 2023^25^	Cohort	Switzerland	136	29	46/119	65.9 ± 13.1	26.8 ± 4.4	Planned for ablation
Racine et al., 2022^9^	Cross‐sectional	France	154	580	308/426	66 ± NR	NR	NR
Pengel et al., 2023^26^	Cross‐sectional	Netherlands	6	170	81/95	40 ± 16.6	NR	Coronary artery disease
Pepplinkhuizen et al., 2022^27^	Cross‐sectional	Netherlands	65	64	15/114	67.16 ± 12.3	28.1 ± 6	Planned for cardioversion
Rajakariar et al., 2020^7^	Cohort	Australia	38	162	58/142	66.3 ± 16.7	NR	University student
Badertscher et al., 2022^8^	Cross‐sectional	Switzerland	34	285	153/166	66.9 ± 3.8	NR	Inpatient of cardiology department
Ford et al., 2022^28^	Cross‐sectional	Australia	22	103	63/62	76 ± 7	29 ± 6	Outpatient of cardiology department
Campo et al., 2022^29^	Cross‐sectional	France	100	162	102/160	67.7 ± 14.8	27.5 ± 5.7	Inpatient/Outpatient without heart implant
Chen et al., 2020^30^	Cross‐sectional	China	150	251	197/204	63.4 ± 14.7	25.5 ± 3.8	Inpatient/Outpatient
Cunha et al., 2020^31^	Cohort	Portugal	45	160	NR	NR	NR	Various settings
Müller et al., 2024^32^	Cohort	Norwegia	18	75	20/73	68 ± 9.9	NR	History of heart valve surgery
Paslı et al., 2024^33^	Cohort	Turkey	180	541	332/389	65 ± 16.3	NR	Emergency unit
Caillol et al., 2021^5^	Cross‐sectional	France	49	207	95/131	66 ± 6	NR	Emergency unit
Velraeds et al., 2023^34^	Cross‐sectional	France	154	569	NR	NR	NR	Inpatient of cardiology department
Scholten et al., 2022^35^	Cross‐sectional	Netherlands	99	121	77/143	70 ± 10	NR	History of cardioversion
Niu et al., 2023^36^	Cohort	China	129	499	282/346	63.8 ± 12.1	NR	Inpatient without heart implant

Abbreviation: NR, not reported.

### 3.3 Quality of the included studies

**FIGURE 2:**
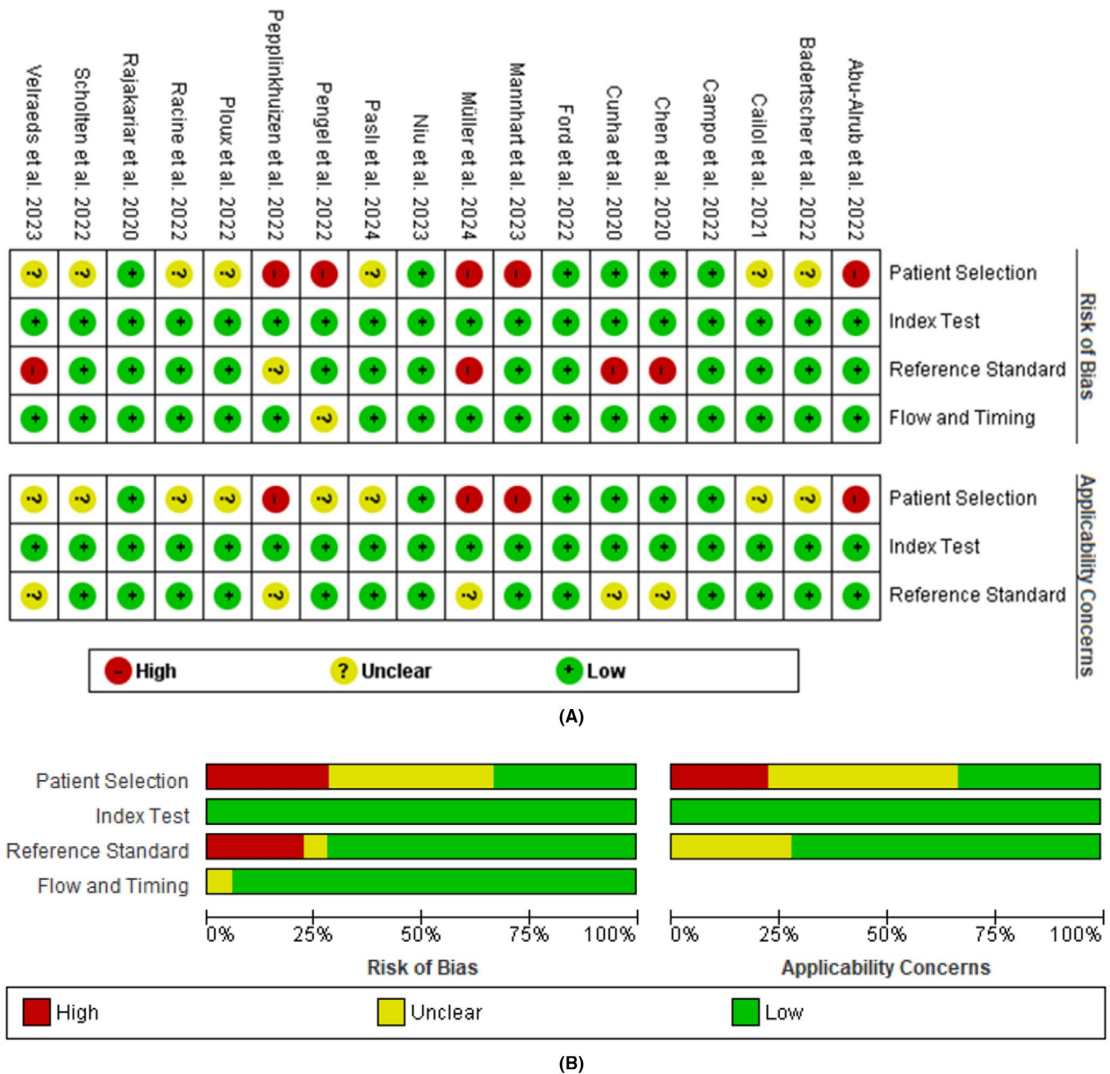
The quality of the studies based on the QUADAS is shown in the detailed “traffic light” plot (A) and the summative plot (B).

The quality of the studies based on the QUADAS‐2 is presented in Figure 2. Five studies were categorized as “high risk” in patient selection, 7 were rated as unclear, and 6 others had a low risk. The high risk in the patient selection domain was attributed to recruiting patients other than those diagnosed with AFib. In the reference standard domain, several studies used only one assessor, which led to them being categorized as high risk.^31^, ^32^, ^37^, ^38^ Another high‐risk study did not report the experience of the medical personnel in interpreting ECGs.^27^ Based on their applicability to address the research question in this systematic review, these studies were categorized as “unclear” in the reference standard assessment. This is because, even if the assessment were carried out accurately by a single reviewer, it would not significantly impact the results of the meta‐analysis. Regarding the flow and timing, only one study failed to report the exact time interval.^27^

### 3.4 Pooled diagnostic values based on algorithmic reading

The forest plot for the sensitivity and specificity of smartwatch ECG in detecting AFib based on the algorithm is presented in Figure S1. The estimated results show that the sensitivity of algorithmic smartwatch ECG reading is 86% (95% CI: 80%–91%). For specificity, the value reaches 94% (95% CI: 89%–97%). Based on the heterogeneity analysis, the *I* ^2^ values for the sensitivity and specificity estimates were 93.29% and 95.78%, respectively. This indicates that the heterogeneity in the sensitivity and specificity meta‐analysis cannot be ignored.

The sensitivity and specificity values were then used to construct the sROC curve, which is presented in Figure S2. The sAUC for the algorithmic ECG reading reached 95% (95% CI: 93%–97%). The analysis continued with the construction of a modifying probability plot, shown in Figure S3. The area under the curve for positive results being larger than the area under the curve for negative test results indicates that the test has a stronger predictive power for detecting a positive condition. The NPV (Negative Predictive Value) and PPV (Positive Predictive Value) for the collective estimates of algorithmic smartwatch ECG reading were 86% (95% CI: 83%–89%) and 93% (95% CI: 90%–96%), respectively.

### 3.5 Pooled diagnostic values based on manual reading

In the manual reading, it was found that the sensitivity of the combined estimate was 96% (95% CI: 94%–97%) (Figure S4). For the specificity of the manual ECG reading, the value reached 95% (95% CI: 92%–96%). The *I* ^2^ analysis indicated high heterogeneity for both sensitivity (I^2^ = 75.42% [95% CI: 65.29%–85.56%]) and specificity (I^2^ = 82.91% [95% CI: 75.07%–88.78%]). The sensitivity and specificity values were then used to construct the sROC curve for identifying AFib through manual ECG reading from the smartwatch (Figure S5). The sAUC value reached 95% (95% CI: 93%–97%). Meanwhile, the modifying probability plot for the manual reading is presented in Figure S6. Unlike the algorithmic reading, the manual reading exhibits a relatively equal area between the curve for positive and negative test results. This indicates that manual ECG reading from the smartwatch shows a high degree of concordance with the 12‐lead ECG in identifying both positive and negative results. The PPV was 95% (95% CI: 93%–97%), with NPV of 94% (95% CI: 92%–96%).

### 3.6 Comparison of smartwatch brands

**FIGURE 3:**
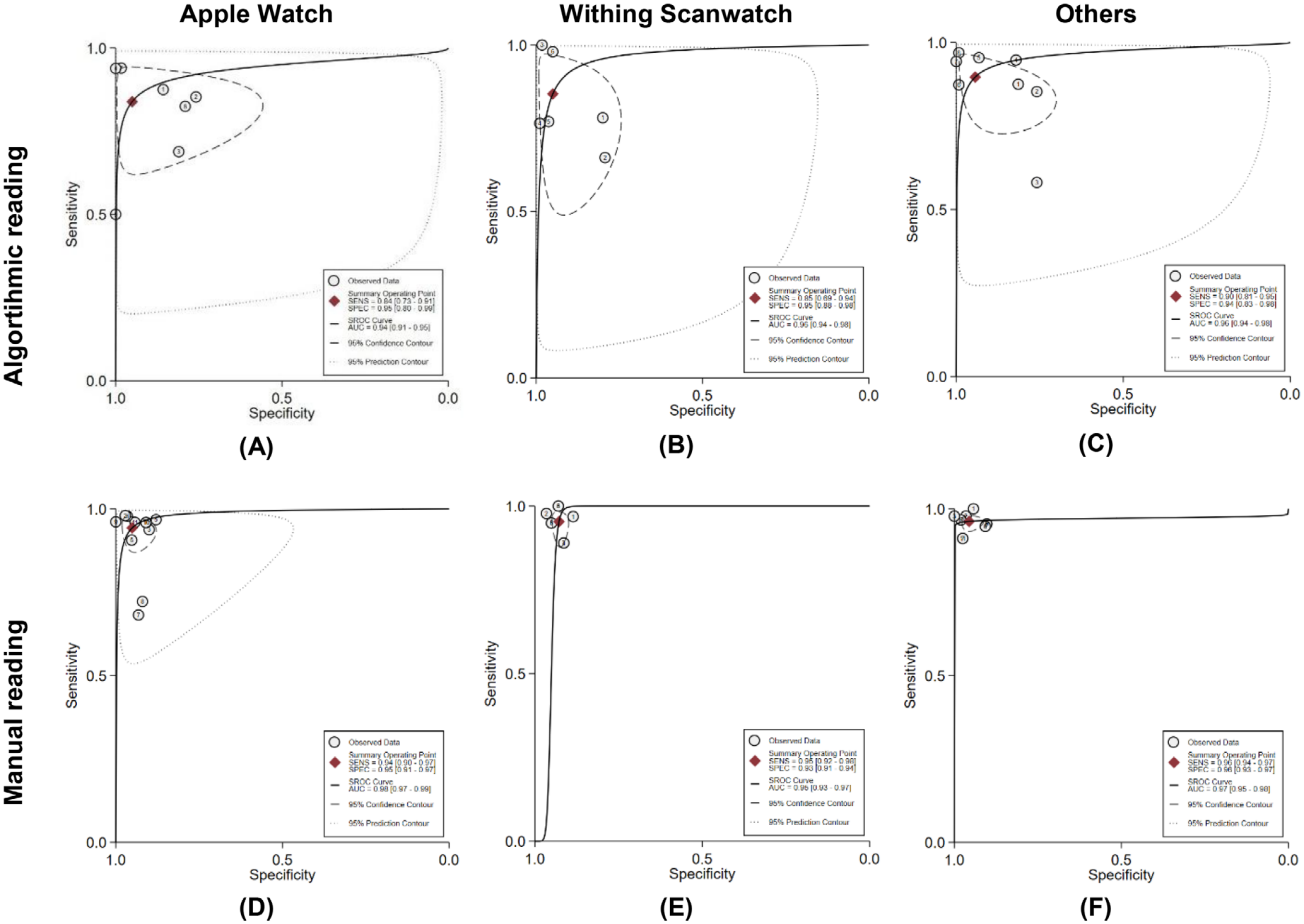
Diagnostic accuracies of different smartwatch brands based on algorithmic and manual readings.

The comparison of diagnostic performance between smartwatch brands based on algorithmic reading is presented in Figure 3. According to the combined estimates, the sensitivity and specificity of the Apple Watch ECG are 84% (95% CI: 73%–91%) and 95% (95% CI: 80%–99%), respectively. The heterogeneity analysis shows *I* ^2^ values for sensitivity and specificity of 92.54% and 96.9%, respectively. In the sROC analysis, the sAUC for Apple Watch reached 94% (95% CI: 91%–95%). The sensitivity and specificity values for Withings Scanwatch were similar, with 85% (95% CI: 69%–94%; *I* ^2^ = 91.93%) and 95% (95% CI: 88%–98%; *I* ^2^ = 92.07%), respectively. For sAUC, Withings Scanwatch (96% [95% CI: 94%–98%]) performed better than the Apple Watch. In the pooled analysis of other brands, such as the Samsung Galaxy Watch, Fitbit Sense, KardiaBand, Amazfit, and Huawei Watch, a higher sensitivity of 90% (95% CI: 81%–95%; *I* ^2^ = 95.18%) was found. As for specificity, the values were similar to those of the Apple Watch and Withings Scanwatch, at 94% (95% CI: 83%–98%; *I* ^2^ = 94.11%). The sAUC for this group was comparable to that of the Withings Scanwatch, reaching 96% (95% CI: 94%–98%).

In manual reading, all three groups had sensitivity and specificity values greater than 90% (Figure 3). The sensitivity and specificity for the Apple Watch ECG in manual reading were 94% (95% CI: 90%–97%) and 95% (95% CI: 91%–97%), with an sAUC value of 98% (95% CI: 97%–99%). The *I* ^2^ values for sensitivity and specificity were 82.74% and 88.59%. Diagnostic performance analysis of the Withings Scanwatch in manual reading shows sensitivity and specificity were 95% (95% CI: 92%–98%) and 93% (95% CI: 91%–94%). This group showed significant heterogeneity for sensitivity (*I* ^2^ = 62.22%), but no significant heterogeneity for specificity (*I* ^2^ = 0.00%). The sAUC for manual reading of the Withings Scanwatch was 95% (95% CI: 93%–97%). For the combined analysis of other brands, sensitivity and specificity were 96% (95% CI: 94%–97%) and 96% (95% CI: 93%–97%). The sAUC was 97% (95% CI: 95%–98%). There was no significant heterogeneity for sensitivity in this group (*I* ^2^ = 0.00%), but significant heterogeneity was observed for specificity (*I* ^2^ = 76.09%).

### 3.7 Results from meta‐regression

The identification of sources of heterogeneity or significant covariates in detecting AFib through algorithmic reading was performed using meta‐regression, with the results summarized in Table 2. The analysis found that heterogeneity in sensitivity estimates was influenced by several factors, including the year of study (*p* < 0.01), study design (*p* = 0.01), study location (*p* = 0.03), smartwatch brand (*p* = 0.01), and the position of smartwatch usage (*p* = 0.01). For specificity, heterogeneity was associated with the year of study (*p* < 0.01), study design (*p* < 0.01), and the position of smartwatch usage (*p* = 0.01). Stratification by study location (*I* ^2^ = 0%; *p*‐Het = 0.98) and the position of smartwatch usage (*I* ^2^ = 0%; *p*‐Het = 0.38) showed that the heterogeneity in these groups could be disregarded.

**TABLE 2:** Results of the meta‐regression for algorithmic detection of AFib based on smartwatch ECG.

Variable	Data, *n*	Diagnostic performance				Heterogenity	
		Sensitivity (95% CI)	*p*‐value	Specificity (95% CI)	*p*‐value	*I* ^2^ (%)	*p*‐Het
Year of study							
≥2023	7	0.77 (0.65–0.90)	Ref.	0.78 (0.60–0.95)	Ref.	80	0.01
<2023	18	0.88 (0.83–0.93)	<0.01	0.96 (0.93–0.98)	<0.01		
Study design							
Cohort	8	0.82 (0.72–0.93)	Ref.	0.84 (0.71–0.98)	Ref.	55	0.11
Cross‐sectional	17	0.87 (0.81–0.92)	0.01	0.96 (0.89–0.99)	<0.01		
Study location							
Europe	18	0.85 (0.79–0.89)	Ref.	0.94 (0.89–0.98)	Ref.	0	0.98
Non‐Europe	7	0.86 (0.77–0.95)	0.03	0.93 (0.86–1.00)	0.26		
Brand							
Apple	12	0.84 (0.75–0.93)	Ref.	0.94 (0.88–1.00)	Ref.	0.90	<0.01
Non‐Apple	13	0.86 (0.75–0.90)	0.01	0.93 (0.88–0.98)	0.32		
Position							
Left Wrist	13	0.83 (0.74–0.91)	Ref.	0.91 (0.84–0.98)	Ref.	0	0.38
Others	12	0.88 (0.82–0.94)	<0.01	0.95 (0.92–0.99)	0.01		
Number of Assessors							
Two assessors	19	0.87 (0.82–0.92)	Ref.	0.93 (0.88–0.98)	Ref.	90	<0.01
One assessor	5	0.82 (0.71–0.94)	0.19	0.96 (0.92–1.00)	0.11		
Gender	25	0.75 (0.30–0.95)	0.52	0.99 (0.93–1.00)	0.06	94	<0.01
Age	25	0.85 (0.78–0.90)	0.91	0.94 (0.88–0.97)	0.99	94	<0.01
BMI	25	0.80 (0.64–0.90)	0.99	0.97 (0.86–1.00)	0.60	99	<0.01

The meta‐regression results for diagnostic performance of AFib through manual reading of smartwatch ECG are presented in Table 3. Regarding sensitivity, the sources of heterogeneity were found to be associated with the year of study, study design, location, brand, usage position, and number of assessors (*p* < 0.01 for each covariate). For specificity, heterogeneity sources were suspected to arise from study design (*p* = 0.02), study location (*p* < 0.01), smartwatch brand (*p* < 0.01), smartwatch usage position (*p* < 0.01), and number of assessors (*p* < 0.01). Nonheterogeneous data groups were successfully obtained based on stratification using study location (*I* ^2^ = 0%; *p*‐Het: 0.5), smartwatch brand (*I* ^2^ = 24%; *p*‐Het: 0.27), usage position (*I* ^2^ = 0%; *p*‐Het: 0.59), and gender (*I* ^2^ = 0%; *p*‐Het >0.99).

**TABLE 3:** Results of the meta‐regression for manual detection of AFib based on smartwatch ECG.

Variable	Data, *n*	Diagnostic performance				Heterogenity	
		Sensitivitity (95% CI)	*p*‐value	Spesificity (95% CI)	*p*‐value	*I* ^2^ (%)	*p*‐Het
Year of study							
≥2023	7	0.96 (0.94–0.99)	Ref.	0.98 (0.97–1.00)	Ref.	80	0.01
<2023	18	0.95 (0.93–0.97)	<0.01	0.93 (0.91–0.95)	0.27		
Study design							
Cohort	8	0.96 (0.94–0.99)	Ref.	0.97 (0.95–0.99)	Ref.	62	0.07
Cross‐Sectional	17	0.95 (0.93–0.97)	<0.01	0.93 (0.91–0.96)	0.02		
Study location							
Europe	18	0.96 (0.94–0.98)	Ref.	0.95 (0.93–0.97)	Ref.	0	0.5
Non‐Europe	7	0.94 (0.91–0.98)	<0.01	0.93 (0.89–0.97)	<0.01		
Merek							
Apple	12	0.94 (0.91–0.97)	Ref.	0.95 (0.92–0.97)	Ref.	24	0.27
Non‐Apple	13	0.97 (0.95–0.99)	<0.01	0.95 (0.92–0.97)	<0.01		
Position							
Left wrist	13	0.96 (0.94–0.98)	Ref.	0.94 (0.91–0.97)	Ref.	0	0.59
Others	12	0.95 (0.93–0.98)	<0.01	0.95 (0.93–0.98)	<0.01		
Number of assessors							
Two assessors	19	0.96 (0.93–0.98)	Ref.	0.95 (0.93–0.97)	Ref.	74	0.02
One assessor	5	0.95 (0.92–0.99)	<0.01	0.93 (0.88–0.98)	<0.01		
Gender	25	0.96 (0.79–0.99)	0.98	0.95 (0.79–0.99)	0.99	0	>0.99
Age	25	0.96 (0.94–0.97)	0.53	0.95 (0.93–0.96)	0.9	76	0.02
BMI	25	0.89 (0.82–0.94)	0.05	0.93 (0.90–0.95)	0.15	99	<0.01

### 3.8 “Uncertain” reading in algorithmic classification of

The percentage of inconclusive results from the algorithmic detection of AFib using the smartwatch ECG was found to be 15% (95% CI: 11%–20%) (Figure S7). Cook's distance analysis identified Niu et al. (2023) as an outlier (<0.19). The estimation was then recalculated after excluding Niu et al. (2023), and the results are shown in Figure S8. The percentage of inconclusive results increased to 17% (95% CI: 14%–20%). When subgroup analysis was performed based on the brand of smartwatch, the rate of inconclusive results was found to be 20% (95% CI: 15%–25%), 16% (95% CI: 11%–22%), and 14% (95% CI: 9%–20%) for Apple Watch, Withings Scanwatch, and others (Samsung Galaxy Watch, Amazfit, KardiaBand, and Fitbit Sense), respectively (Figure S9).

The Apple Watch algorithm is unable to read the ECG results when the heart rate (HR) is >150 bpm or <50 bpm. On the Samsung Galaxy Watch, inconclusive results are observed when the patient's HR is <50 bpm or >120 bpm. Meanwhile, Withings Scanwatch uses HR <50 bpm or >100 bpm as criteria to exclude the algorithmic reading. Other contributing factors include poor recording quality, noise, and the presence of artifacts, which can lead to changes in the QRS complex.

### 3.9 Manual interpretability of smartwatch ECG

The rate of results that are difficult or impossible to interpret manually was found to be 5% (95% CI: 2%–9%) (Figure S10). However, in the combined estimate, an outlier was identified from Abu‐Alrub et al., 2022^6^ with a Cook's distance >0.31. After excluding this value, the combined rate was found to be 3% (95% CI: 1%–6%) (Figure S11). A comparison between Apple Watch and Withings Scanwatch is presented in Figure S12. The rate of ECGs that could not be manually interpreted for Apple Watch (6% [95% CI: 4%–9%]) was higher compared to Withings Scanwatch (2% [95% CI: 0%–6%]). For the interpretability of manual readings, Cohen's Kappa (*κ*) values were used. The analysis revealed a *κ* value of 0.83 (95% CI: 0.73–0.90) (Figure S13). The Cook's distance for each study did not exceed the threshold (<0.4). Difficult or unreadable manual readings were because of several reasons such as noise, motion artefacts, and baseline wanders (Table S4).

### 3.10 Publication bias

Publication bias in the diagnostic accuracy meta‐analysis can be seen in Figure S14. Based on the asymmetry test, publication bias was found in the analysis for manual smartwatch ECG readings (*p* = 0.04). For the analysis of the interpretability parameters of the smartwatch ECG, publication bias identification was performed based on the funnel plot (Figure S15). Based on Egger's correlation, significant publication bias was observed in the pooled proportion estimates for algorithmic reading with *p*‐Egg = 0.014.

## 4 DISCUSSION

The present meta‐analysis provides novel insights into the diagnostic performance and interpretability of smartwatch‐based ECGs for AFib detection. The findings confirm that these devices offer high diagnostic accuracy, particularly when ECGs are interpreted manually by trained personnel. Clinically, smartwatch ECGs may help identify AFib in patients with intermittent symptoms, supporting earlier diagnosis and timely intervention. From a public health perspective, their ease of use makes them suitable for integration into community‐based screening programs, especially in settings with limited access to standard 12‐lead ECGs. Moreover, this present study is among the first to quantify the proportion of unreadable or inconclusive smartwatch ECGs, providing a benchmark for future algorithm improvements and device enhancements. This information is valuable for both clinicians and developers, especially when aiming for large‐scale or unsupervised use of this technology.

### 4.1 Diagnostic performance

The results of this study show that the smartwatch ECG has a sensitivity of 86% and a specificity of 94% in detecting AFib through digital logarithmic detection. When the smartwatch ECG is read manually, its sensitivity increases to 96%. The specificity in manual reading (95%) is not much different compared to algorithmic reading. The accuracy measured through sAUC shows a value of 95%, both for algorithmic and manual readings. The increase in sensitivity with manual reading aligns with several studies included in this systematic review.^6^, ^8^, ^9^, ^25^, ^28^, ^35^, ^38^ Compared to the sensitivity of algorithmic reading in this study, a meta‐analysis conducted in 2020 estimated that the sensitivity of smartwatches reached 94%.^39^ This indicates that studies in the following years, with different settings and populations, provide a clearer picture of the accuracy of these devices. Previous meta‐analyses reported that artificial intelligence algorithms can use both PPG and ECG data with sensitivity and specificity rates above 90%.^11^ Other meta‐analyses have even shown that the sensitivity of smartwatches in detecting non‐specific arrhythmias can reach 100%, with specificity and accuracy rates of 95% and 97%, respectively.^40^

A previous report suggests that diagnostic values can be categorized into high, medium, and low, with ranges of >90%, 70–90%, and <70%, respectively.^41^ Thus, the algorithmic detection of AFib in the present study can be classified as having moderate sensitivity but high specificity. Meanwhile, in manual reading, both sensitivity and specificity can be categorized as high. This indicates that negative results from the algorithmic smartwatch ECG reading can be trusted. However, positive results from the smartwatch ECG still require manual interpretation by a cardiologist or a competent healthcare professional. A previous study in the UK reported that the average time required to detect heart rhythms associated with symptoms decreased from 42.9 to 9.5 days.^42^ In line with the results of this study, the smartwatch ECG shows high accuracy when manually read, making it a significant modality for clinical decision‐making. Previous systematic reviews have also concluded that the use of health monitoring devices, such as smartwatches, results in better healthcare outcomes.^43^ However, the increase in the number of visits and patient anxiety needs to be anticipated as a consequence of the risk of false‐positive results. It is also worth noting that smartwatch ECGs are not a direct replacement for Holter monitors since they work by acquiring continuous ECG over 24–48 h or longer. Meanwhile, smartwatch ECGs rely on user‐initiated recordings.

Herein, year of the study, study location, and smartwatch brand are three covariates that are interconnected, particularly in terms of technology and digital algorithms. The study year is closely related to the watch brand used. For example, KardiaBand first released the ECG feature, but it had to be paired with the Apple Watch. Before 2023, the ECG feature on smartwatches was generally still the first generation, such as the Apple Watch Series 4, which was first released in 2018, or the Samsung Galaxy Watch in 2021.^44^, ^45^ In the first generation, the technology was generally still underdeveloped and required many improvements. Studies conducted in 2023 and beyond used more advanced technology and algorithms in detecting AFib, resulting in higher sensitivity and specificity compared to previous years. The same applies to the location; for example, studies on the Withings Scanwatch and Apple Watch were largely dominated by European countries.

Based on the meta‐regression, it was found that the estimated diagnostic values tend to be lower in cohort studies compared to cross‐sectional studies. This is because cohort studies generally require a longer follow‐up period, which results in greater variation in detection outcomes because of changes in the participants' conditions over time. Additionally, cohort studies involve more diverse settings, which may reduce sensitivity and specificity compared to cross‐sectional studies that are usually more controlled and focused on a specific point in time.

The impact of the number and type of assessors on the meta‐analysis results in this study was only observed in manual readings. Specificity and sensitivity in studies that used one assessor were lower compared to studies that used two assessors. However, sensitivity was observed to be lower in studies with a single assessor. Using more than one assessor allows for cross‐checking and discussion between assessors, thus reducing the likelihood of errors or biases in the reading. With two assessors, diagnostic decisions become more accurate and verified, which enhances specificity. On the other hand, a single assessor tends to rely on subjective judgment without verification from another party, which can lead to lower sensitivity and an increased likelihood of false‐negative results or other inaccuracies.

Meanwhile, the location of smartwatch usage was found to significantly influence the meta‐analysis results, both in algorithmic and manual readings. The location of smartwatch usage is suspected to affect the quality of the ECG generated, which in turn impacts the readings, whether through algorithms or manually. One study reported that combining ECG recordings from different positions resulted in better diagnostic accuracy compared to recordings taken from the left wrist.^24^ Some studies also determined the placement of the smartwatch based on the dominant hand.^7^, ^8^, ^9^ Body movement and muscle contractions can generate additional electrical signals similar to the heart's electrical flow, known as interference signals or noise. When muscles contract, the small electrical current generated by this muscle activity can mix with the heart's signal, resulting in distortion in the ECG signal recording.

### 4.2 Interpretability

The results of the study show that 17% of the total ECGs recorded by the smartwatch could not be algorithmically classified as either AFib or sinus rhythm. In manual reading, the percentage of ECGs that were unreadable was 3%, with a kappa coefficient of 0.83. The high number of ECGs that cannot be read algorithmically may be one of the reasons why the sensitivity of the device is lower compared to manual reading. In algorithm‐based readings, AFib and non‐AFib conditions are differentiated based on the irregularity of R‐R intervals and heart rate (HR). To avoid false‐negative results, the algorithm compensates by setting minimum and maximum HR values. However, this approach results in AFib cases with tachycardia and bradycardia being undetected or resulting in false negatives.^23^ In addition, to accurately determine the condition of AFib, the P‐wave profile should also be observed over several seconds.^6^

Conditions such as atrial flutter with AV block, premature atrial contractions (PAC), and premature ventricular contractions can cause irregularities in the R‐R interval.^23^ Although atrial flutter typically involves only a fast atrial rhythm without causing irregularity. Additionally, nonpathological conditions like sinus arrhythmia can cause the R‐R interval to vary because of the natural response to breathing (the heart beats faster when inhaling and slows down when exhaling). This was also observed in AFib detection based on tachograms generated by PPG sensors, where sinus arrhythmia is one of the conditions that causes irregular heartbeats.^46^ To address this, a study developed an algorithm capable of detecting whether the irregularity in the R‐R interval is regular or irregular. By identifying R‐R intervals with an “irregularly irregular” pattern, the study was able to significantly improve sensitivity and specificity.^34^

### 4.3 Comparisons of accuracy and interpretability of smartwatch brands

The comparison between different smartwatch brands showed no significant differences in sensitivity and specificity values for algorithmic AFib detection by Apple Watch, Withings Scanwatch, and others. Sensitivity of 90% was only achieved by the combined analysis of Huawei Watch GT2 Pro, AliveCor KardiaBand, Fitbit Sense, Amazfit Health Band, and Samsung Galaxy Watch. Among these five brands, a specificity above 90% was reported by studies using AliveCor KardiaBand and Huawei Watch GT2 Pro.^7^, ^28^, ^36^ Previous research reported that Apple Watch Series 4 had only 50% sensitivity, which increased to 68% with manual interpretation.^28^ The advantage of AliveCor KardiaBand over Apple Watch (especially Series 4) is speculated to be related to the more mature algorithm on AliveCor KardiaBand, which was developed earlier.^7^, ^28^ Algorithm development is common in the technology sector, especially in health monitoring. For example, the ability of PPG to detect AFib continues to improve with the development of algorithms using machine learning approaches.^18^

As previously mentioned, the highest/lowest heart rate (HR) that can be read by Apple Watch, Samsung Galaxy Watch, and Withings Scanwatch are 150/50 bpm, 120/50 bpm, and 100/50 bpm, respectively.^6^ Therefore, Withings Scanwatch automatically excludes the most recordings, followed by Samsung Galaxy and Apple Watch. However, this statement does not align with the results obtained from this meta‐analysis, where Apple Watch had the highest percentage of uncertain results (20%) compared to Withings (16%) and others (14%). This indicates that factors other than heart rate detection limits contribute to the uncertain results on Apple Watch. Factors that may influence this value include signal quality, sensor sensitivity, or the data processing algorithm used. The results in this study also suggest that Apple Watch may be more sensitive to artifacts or external disturbances, which can increase the number of failures in algorithmic interpretation. This observation aligns with previous studies that found Apple Watch algorithms to be sensitive to artifacts generated by muscle contractions and body movements.^47^Additionally, the frequent appearance of low‐energy electrical noise signals in ECG recordings from Apple Watch and Samsung Galaxy Watch has also been reported in previous studies.^6^, ^47^

### 4.4 Strengths and limitations

This study is the most comprehensive and up‐to‐date meta‐analysis on smartwatch ECG, following the first one conducted in 2019.^39^ This study is the first to perform a meta‐analysis on the interpretability of smartwatch ECGs. Another strength of this study is the diagnostic accuracy and interpretability parameters, which were estimated through meta‐analysis, providing estimates based on a larger sample. Meta‐regression and Cook's distance analyses were performed to identify covariates and outlier data, allowing for a more comprehensive understanding of the findings. However, there are some limitations in this study that should be acknowledged. First, the authors did not search for data outside of those reported in peer‐reviewed journals. Although this limited the amount of data collected, it ensures that the data has gone through a thorough evaluation process by experts. Furthermore, caution is needed when interpreting the results of this study, especially when significant heterogeneity is observed. Most included studies focused on specific populations, such as patients with known cardiovascular conditions or those undergoing medical evaluations. Consequently, the generalizability of these findings to young, asymptomatic individuals remains uncertain. Another limitation is the lack of information on software or algorithm versions used in the included studies. Even within the same device brand, different firmware or algorithm updates may affect ECG interpretation performance. However, most studies did not report the specific software or algorithm versions embedded in the smartwatches, limiting our ability to assess their potential influence on diagnostic accuracy.

## 5 CONCLUSION

Smartwatch ECG has a high accuracy in detecting AFib, especially when the interpretation is done manually by medical professionals. The results of this meta‐analysis indicate that, although this technology has limitations, such as sensitivity to motion artifacts and limitations in heart rate range, several brands including Apple Watch and Withings Scanwatch demonstrate high specificity in AFib detection. Smartwatch ECG can function as an initial screening tool for AFib, particularly in high‐risk populations, with positive results requiring further confirmation through clinical examination. The integration of this technology enables healthcare providers to access data in real time, accelerate clinical decision making, and support the implementation of efficient screening programs through portable devices connected to electronic health systems. Further research is needed in low‐risk populations to determine their applicability in general screening. Additionally, further exploration of smartwatch ECG's ability to detect other cardiovascular disorders could be carried out to expand its clinical benefits.

## FUNDING INFORMATION

This study received no external funding.

## CONFLICT OF INTEREST STATEMENT

All authors declare that they have no known conflicts of interest in relation to the publication of this work.

## Supporting information


Data S1.

Data S2.

Data S3.

## References

[1] Mensah G , Fuster V , Murray C , Roth GGBoCDC . Global burden of cardiovascular diseases and risks, 1990‐2022. J Am Coll Cardiol. 2023;82:2350–2473. 38092509 10.1016/j.jacc.2023.11.007 PMC7615984

[2] Lippi G , Sanchis‐Gomar F , Cervellin G . Global epidemiology of atrial fibrillation: an increasing epidemic and public health challenge. Int J Stroke. 2021;16:217–221. 31955707 10.1177/1747493019897870

[3] Cai W , Chen Y , Guo J , Han B , Shi Y , Ji L , et al. Accurate detection of atrial fibrillation from 12‐lead ECG using deep neural network. Comput Biol Med. 2020;116:103378. 31778896 10.1016/j.compbiomed.2019.103378

[4] Wong KC . Atrial Fibrillation Screening in the Community: An Integrated Continual Care Approach. 2024 In, The University of Sydney.

[5] Caillol T , Strik M , Ramirez FD , Abu‐Alrub S , Marchand H , Buliard S , et al. Accuracy of a smartwatch‐derived ECG for diagnosing Bradyarrhythmias, tachyarrhythmias, and cardiac ischemia. Circ Arrhythm Electrophysiol. 2021;14:E009260. 33441002 10.1161/CIRCEP.120.009260

[6] Abu‐Alrub S , Strik M , Ramirez FD , Moussaoui N , Racine HP , Marchand H , et al. Smartwatch electrocardiograms for automated and manual diagnosis of atrial fibrillation: a comparative analysis of three models. Front Cardiovasc Med. 2022;9:836375. 35187135 10.3389/fcvm.2022.836375 PMC8854369

[7] Rajakariar K , Koshy AN , Sajeev JK , Nair S , Roberts L , Teh AW . Accuracy of a smartwatch based single‐lead electrocardiogram device in detection of atrial fibrillation. Heart. 2020;106:665–670. 31911507 10.1136/heartjnl-2019-316004

[8] Badertscher P , Lischer M , Mannhart D , Knecht S , Isenegger C , de Lavallaz JDF , et al. Clinical validation of a novel smartwatch for automated detection of atrial fibrillation. Heart Rhythm. 2022;3:208. 10.1016/j.hroo.2022.02.004 PMC9043399 35496455

[9] Racine H‐P , Strik M , van der Zande J , Alrub SA , Caillol T , Haïssaguerre M , et al. Role of coexisting ECG anomalies in the accuracy of smartwatch ECG detection of atrial fibrillation. Can J Cardiol. 2022;38:1709–1712. 36334937 10.1016/j.cjca.2022.08.222

[10] Vetta G , Magnocavallo M , Parlavecchio A , Caminiti R , Polselli M , Sorgente A , et al. Diagnostic accuracy of smartwatch in detecting atrial fibrillation: a systemic review and meta‐analysis. Europace. 2023;25:euad122‐646.

[11] Manetas‐Stavrakakis N , Sotiropoulou IM , Paraskevas T , Maneta Stavrakaki S , Bampatsias D , Xanthopoulos A , et al. Accuracy of artificial intelligence‐based Technologies for the Diagnosis of atrial fibrillation: a systematic review and meta‐analysis. J Clin Med. 2023;12:6576. 37892714 10.3390/jcm12206576 PMC10607777

[12] Shahid S , Iqbal M , Saeed H , Hira S , Batool A , Khalid S , et al. Diagnostic accuracy of apple watch electrocardiogram for atrial fibrillation: a systematic review and meta‐analysis. JACC Cardiovasc Interv. 2025;4:101538. 10.1016/j.jacadv.2024.101538 PMC11780081 39886315

[13] Whiting PF , Rutjes AW , Westwood ME , Mallett S , Deeks JJ , Reitsma JB , et al. QUADAS‐2: a revised tool for the quality assessment of diagnostic accuracy studies. Ann Intern Med. 2011;155:529–536. 22007046 10.7326/0003-4819-155-8-201110180-00009

[14] Evans HJ , Gibson NA , Bennett J , Chan SY , Gavlak J , Harman K , et al. British Thoracic Society guideline for diagnosing and monitoring paediatric sleep‐disordered breathing. Thorax. 2023;78:s1–s27. 10.1136/thorax-2022-218938 37295792

[15] Iqhrammullah M , Refin RY , Andika FF , Amirah S , Abdurrahman MF , Alina M , et al. Dropout rate in clinical trials of smartphone apps for diabetes management: a meta‐analysis. Diabetes Res Clin Pract. 2024;212:111723. 38830484 10.1016/j.diabres.2024.111723

[16] Bumgarner JM , Lambert CT , Hussein AA , Cantillon DJ , Baranowski B , Wolski K , et al. Smartwatch algorithm for automated detection of atrial fibrillation. J Am Coll Cardiol. 2018;71:2381–2388. 29535065 10.1016/j.jacc.2018.03.003

[17] Yalin K , Soysal AU , Ikitimur B , Yabaci BI , Onder SE , Atici A , et al. Diagnostic accuracy of apple watch series 6 recorded single‐lead ECGs for identifying supraventricular tachyarrhythmias: a comparative analysis with invasive electrophysiological study. J Interv Card Electrophysiol. 2023;67:1145–1151. 37985539 10.1007/s10840-023-01695-6

[18] Tison GH , Sanchez JM , Ballinger B , Singh A , Olgin JE , Pletcher MJ , et al. Passive detection of atrial fibrillation using a commercially available smartwatch. JAMA Cardiol. 2018;3:409–416. 29562087 10.1001/jamacardio.2018.0136 PMC5875390

[19] Leroux J , Strik M , Ramirez FD , Ploux S , Sacristan B , Chabaneix‐Thomas J , et al. Using a smartwatch to record an electrocardiogram in the pediatric population. J Electrocardiol. 2022;71:25–27. 35016137 10.1016/j.jelectrocard.2021.12.009

[20] Koshy AN , Sajeev JK , Nerlekar N , Brown AJ , Rajakariar K , Zureik M , et al. Smart watches for heart rate assessment in atrial arrhythmias. Int J Cardiol. 2018;266:124–127. 29887428 10.1016/j.ijcard.2018.02.073

[21] Duncker D , Ding WY , Etheridge S , Noseworthy PA , Veltmann C , Yao X , et al. Smart wearables for cardiac monitoring—real‐world use beyond atrial fibrillation. Sensors. 2021;21:2539. 33916371 10.3390/s21072539 PMC8038592

[22] Chon KH , McManus DD . Detection of atrial fibrillation using a smartwatch. Nat Rev Cardiol. 2018;15:657–658. 29985453 10.1038/s41569-018-0057-1 PMC6324540

[23] Strik M , Ploux S , Ramirez FD , Abu‐Alrub S , Jaîs P , Haïssaguerre M , et al. Smartwatch‐based detection of cardiac arrhythmias: beyond the differentiation between sinus rhythm and atrial fibrillation. Heart Rhythm. 2021;18:1524–1532. 34147700 10.1016/j.hrthm.2021.06.1176

[24] Ploux S , Strik M , Caillol T , Ramirez FD , Abu‐Alrub S , Marchand H , et al. Beyond the wrist: using a smartwatch electrocardiogram to detect electrocardiographic abnormalities. Arch Cardiovasc Dis. 2022;115:29–36. 34953753 10.1016/j.acvd.2021.11.003

[25] Mannhart D , Lefebvre B , Gardella C , Henry C , Serban T , Knecht S , et al. Clinical validation of an artificial intelligence algorithm offering cross‐platform detection of atrial fibrillation using smart device electrocardiograms. Arch Cardiovasc Dis. 2023;116:249–257. 37183163 10.1016/j.acvd.2023.04.003

[26] Pengel LKD , Robbers‐Visser D , Groenink M , Winter MM , Schuuring MJ , Bouma BJ , et al. A comparison of ECG‐based home monitoring devices in adults with CHD. Cardiol Young. 2023;33:1129–1135. 35844104 10.1017/S1047951122002244

[27] Pepplinkhuizen S , Hoeksema WF , van der Stuijt W , van Steijn NJ , Winter MM , Wilde AAM , et al. Accuracy and clinical relevance of the single‐lead apple watch electrocardiogram to identify atrial fibrillation. Cardiovasc Digit Health J. 2022;3:S17–S22. 36589758 10.1016/j.cvdhj.2022.10.004 PMC9795256

[28] Ford C , Xie CX , Low A , Rajakariar K , Koshy AN , Sajeev JK , et al. Comparison of 2 SMART watch algorithms for detection of atrial fibrillation and the benefit of clinician interpretation: SMART WARS study. JACC Clin Electrophysiol. 2022;8:782–791. 35738855 10.1016/j.jacep.2022.02.013

[29] Campo D , Elie V , de Gallard T , Bartet P , Morichau‐Beauchant T , Genain N , et al. Atrial fibrillation detection with an analog smartwatch: prospective clinical study and algorithm validation. JMIR Form Res. 2022;6:e37280. 35481559 10.2196/37280 PMC9675016

[30] Chen E , Jiang J , Su R , Gao M , Zhu S , Zhou J , et al. A new smart wristband equipped with an artificial intelligence algorithm to detect atrial fibrillation. Heart Rhythm. 2020;17:847–853. 32354449 10.1016/j.hrthm.2020.01.034

[31] Cunha S , Antunes E , Antoniou S , Tiago S , Relvas R , Fernandez‐Llimós F , et al. Raising awareness and early detection of atrial fibrillation, an experience resorting to mobile technology centred on informed individuals. Res Soc Adm Pharm. 2020;16:787–792. 10.1016/j.sapharm.2019.08.036 31473110

[32] Müller M , Hanssen TA , Johansen D , Jakobsen Ø , Pedersen JE , Aamot Aksetøy IL , et al. Validity of a smartwatch for detecting atrial fibrillation in patients after heart valve surgery: a prospective observational study. Scand Cardiovasc J. 2024;58:2353069. 38794854 10.1080/14017431.2024.2353069

[33] Paslı S , Topçuoğlu H , Yılmaz M , Yadigaroğlu M , İmamoğlu M , Karaca Y . Diagnostic accuracy of apple watch ECG outputs in identifying dysrhythmias: a comparison with 12‐Lead ECG in emergency department. Am J Emerg Med. 2024;79:25–32. 38330880 10.1016/j.ajem.2024.01.046

[34] Velraeds A , Strik M , van der Zande J , Fontagne L , Haissaguerre M , Ploux S , et al. Improving automatic smartwatch electrocardiogram diagnosis of atrial fibrillation by identifying regularity within irregularity. Sensors. 2023;23:9283. 38005669 10.3390/s23229283 PMC10674836

[35] Scholten J , Jansen WPJ , Horsthuis T , Mahes AD , Winter MM , Zwinderman AH , et al. Six‐lead device superior to single‐lead smartwatch ECG in atrial fibrillation detection. Am Heart J. 2022;253:53–58. 35850242 10.1016/j.ahj.2022.06.010

[36] Niu Y , Wang H , Wang H , Zhang H , Jin Z , Guo Y . Diagnostic validation of smart wearable device embedded with single‐lead electrocardiogram for arrhythmia detection. Digit Health. 2023;9:20552076231198682. 37667685 10.1177/20552076231198682 PMC10475230

[37] Pepplinkhuizen S , Hoeksema WF , van der Stuijt W , Smeding L , Wilde AA , Knops RE . Accuracy of the apple watch electrocardiogram to detect atrial fibrillation in a real‐world clinical setting. Circulation. 2021;144:A13805. 10.1016/j.cvdhj.2022.10.004 PMC9795256 36589758

[38] Chen Y , Huang QF , Sheng CS , Zhang W , Shao S , Wang D , et al. Detection rate and treatment gap for atrial fibrillation identified through screening in community health centers in China (AF‐CATCH): a prospective multicenter study. PLoS Med. 2020;17:e1003146. 32673305 10.1371/journal.pmed.1003146 PMC7365395

[39] Prasitlumkum N , Cheungpasitporn W , Chokesuwattanaskul A , Thangjui S , Thongprayoon C , Bathini T , et al. Diagnostic accuracy of smart gadgets/wearable devices in detecting atrial fibrillation: a systematic review and meta‐analysis. Arch Cardiovasc Dis. 2021;114:4–16. 32921618 10.1016/j.acvd.2020.05.015

[40] Nazarian S , Lam K , Darzi A , Ashrafian H . Diagnostic accuracy of smartwatches for the detection of cardiac arrhythmia: systematic review and meta‐analysis. J Med Internet Res. 2021;23:e28974. 34448706 10.2196/28974 PMC8433941

[41] Young Ho L . Overview of the process of conducting meta‐analyses of the diagnostic test accuracy. J Rheumatol. 2018;25:3–10.

[42] Reed MJ , Grubb NR , Lang CC , O'brien R , Simpson K , Padarenga M , et al. Multi‐centre randomised controlled trial of a smartphone‐based event recorder alongside standard care versus standard care for patients presenting to the emergency department with palpitations and pre‐syncope: the IPED (investigation of palpitations in the ED) study. EClinicalMed. 2019;8:37–46. 10.1016/j.eclinm.2019.02.005 PMC6537555 31193636

[43] Triantafyllidis A , Kondylakis H , Katehakis D , Kouroubali A , Alexiadis A , Segkouli S , et al. Smartwatch interventions in healthcare: a systematic review of the literature. Int J Med Inform. 2024;190:105560. 39033723 10.1016/j.ijmedinf.2024.105560

[44] Apple Inc . Apple Watch Series 4—Spesifikasi Teknis. 2018.

[45] Samsung . Galaxy Watch4 and Galaxy Watch4 Classic: Reshaping the Smartwatch Experience. 2021.

[46] Perino AC , Gummidipundi SE , Lee J , Hedlin H , Garcia A , Ferris T , et al. Arrhythmias other than atrial fibrillation in those with an irregular pulse detected with a smartwatch: findings from the apple heart study. Circ Arrhythm Electrophysiol. 2021;14:e010063. 34565178 10.1161/CIRCEP.121.010063

[47] Saghir N , Aggarwal A , Soneji N , Valencia V , Rodgers G , Kurian T . A comparison of manual electrocardiographic interval and waveform analysis in lead 1 of 12‐lead ECG and apple watch ECG: a validation study. Cardiovasc Digit Health J. 2020;1:30–36. 35265871 10.1016/j.cvdhj.2020.07.002 PMC8890353

