# Supplementary material for: Accuracy and interpretability of smartwatch electrocardiogram for early detection of atrial fibrillation: A systematic review and meta‐analysis
Source: J Arrhythm. 2025 May 22;41(3):e70087. doi: 10.1002/joa3.70087 (PMC12096014; doi:10.1002/joa3.70087)
Supplement: Supplementary file 2 — Data S2. [file JOA3-41-e70087-s002.docx]

Figure S1. Forest plot for the sensitivity and specificty of smartwatch ECG in AFib identification based on algorithmic readings.

Figure S2. sROC plot of smartwatch ECG in AFib identification based on algorithmic readings.

Figure S3. Probability modifying plot of smartwatch ECG in AFib identification based on algorithmic readings.

Figure S4. Forest plot for the sensitivity and specificty of smartwatch ECG in AFib identification based on manual readings.

Figure S5. sROC plot of smartwatch ECG in AFib identification based on manual readings.

Figure S6. Probability modifying plot of smartwatch ECG in AFib identification based on algorithmic readings.


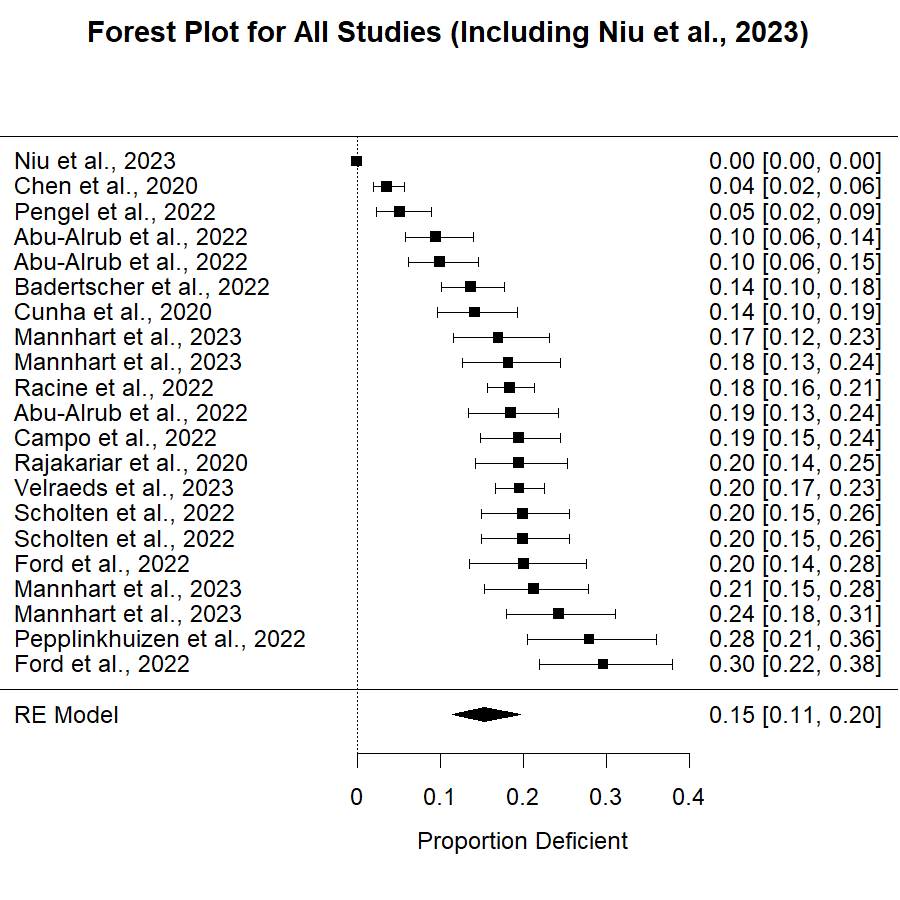


(a)


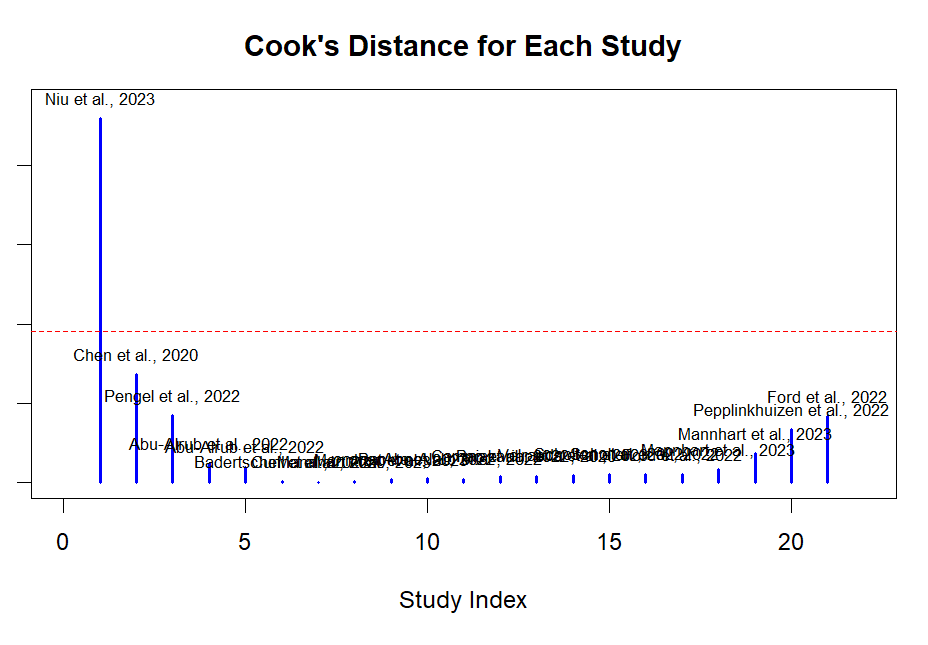


(b)

Figure S7. Forest plot (a) and Cook’s distance plot (b) for uncertain result in discriminating AFib and Non-AFib by smartwatch ECG.


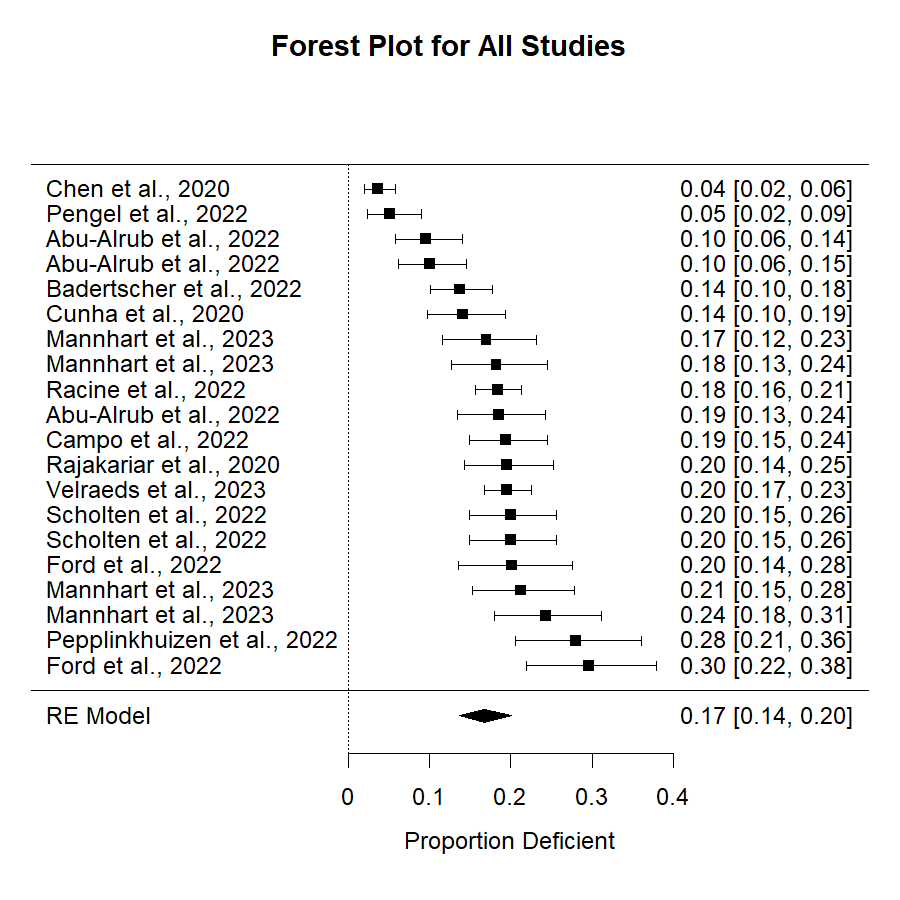


Figure S8. Forest plot (a) and Cook’s distance plot (b) for uncertain results in discriminating AFib and Non-AFib by smartwatch ECG excluding Niu et al., 2023 (outlier).

| 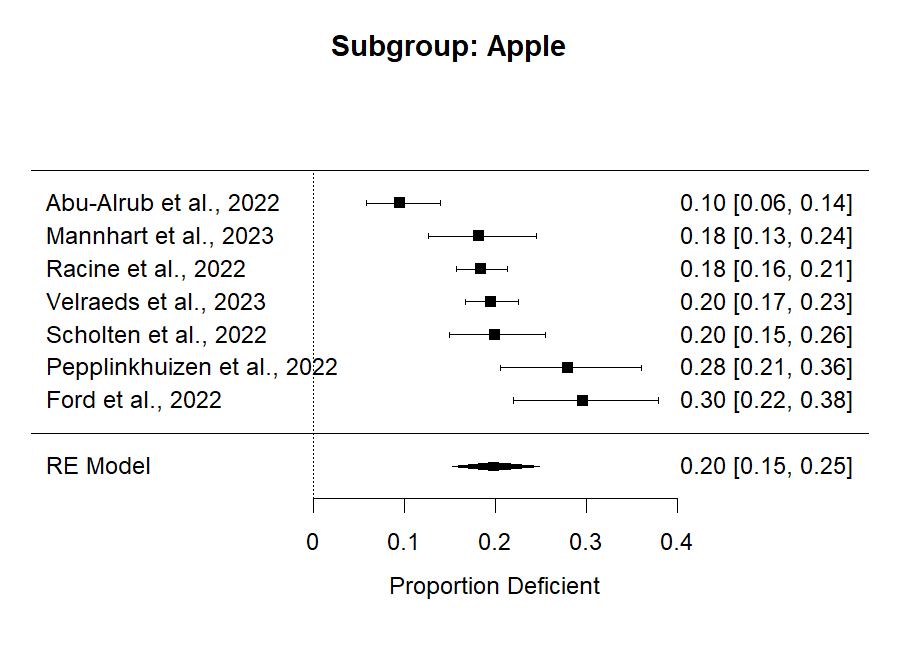  (a) | 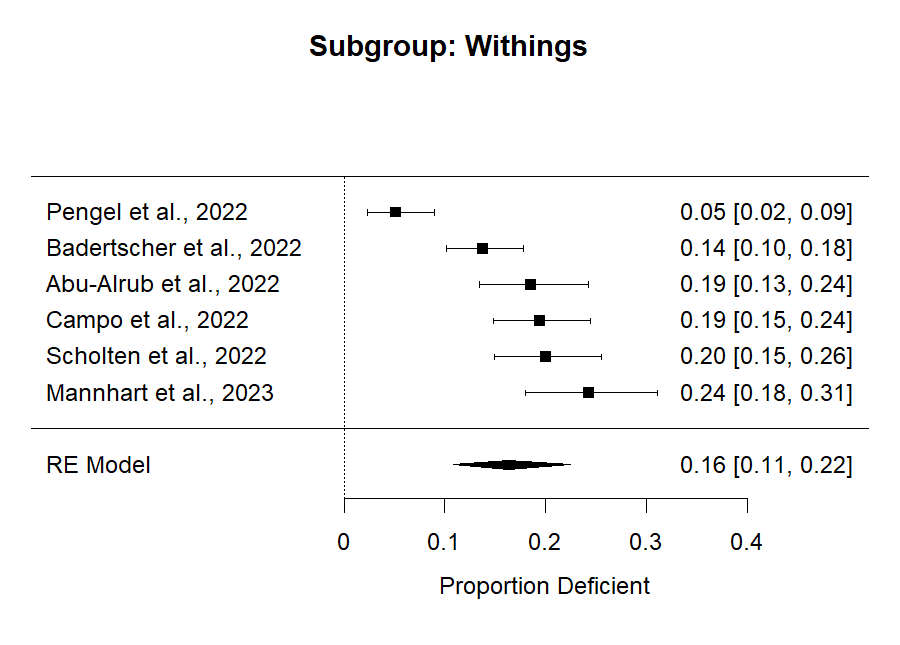  (b) |
| --- | --- |
| 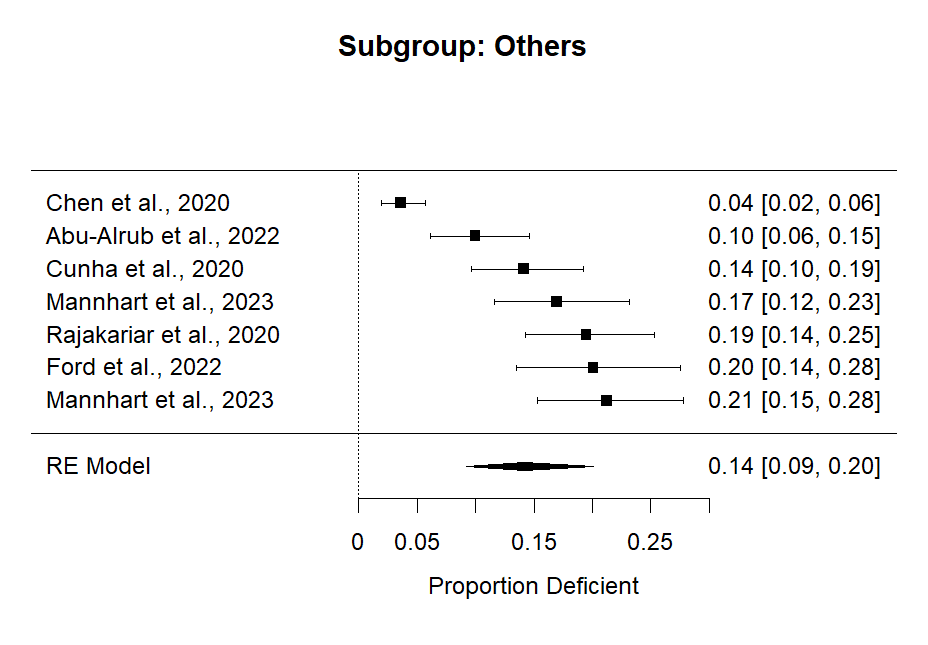  (c) | |

Figure S9. Forest plot for the percentage of uncertain results in discriminating AFib and Non-AFib by Apple Watch (a), Withing Scanwatch (b) and others (c).


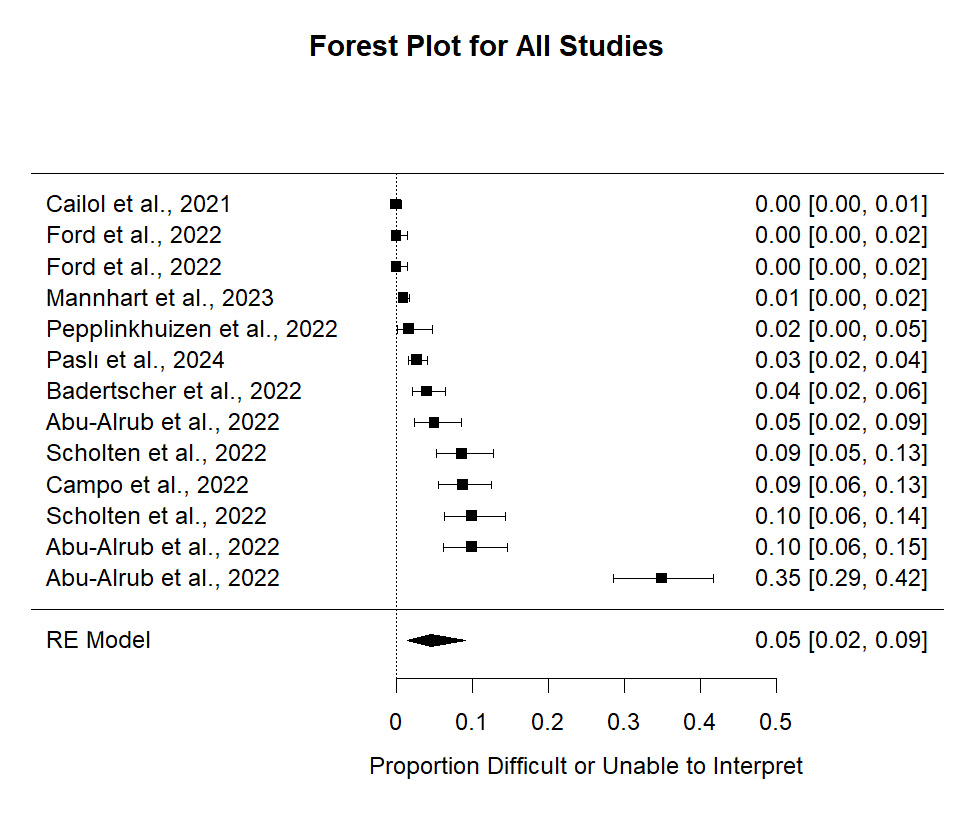


(a)


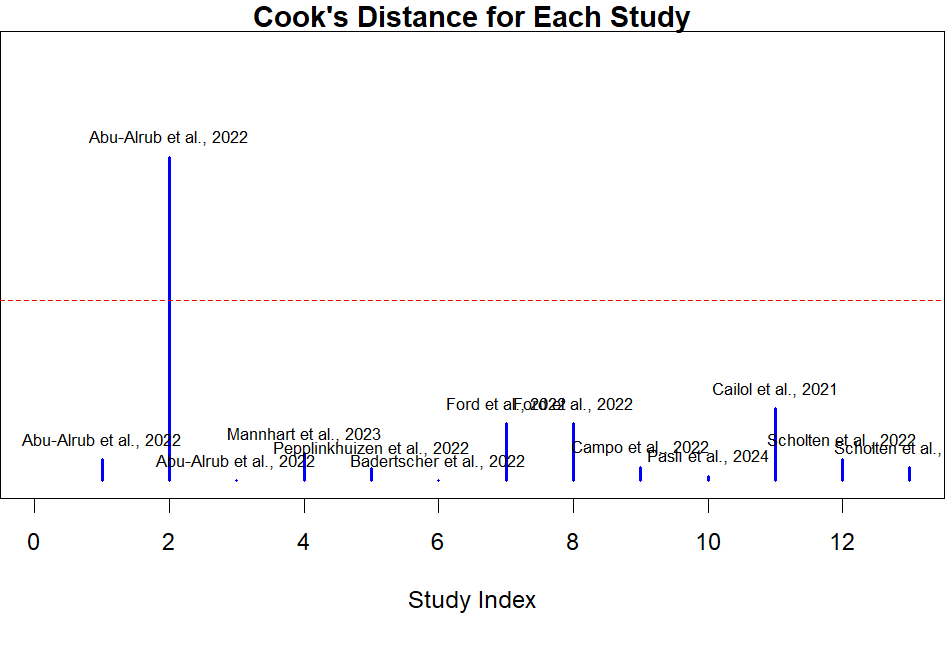


(b)

Figure S10. Forest plot (a) dan Cook’s distance plot (b) for non-readable smartwatch ECGs .


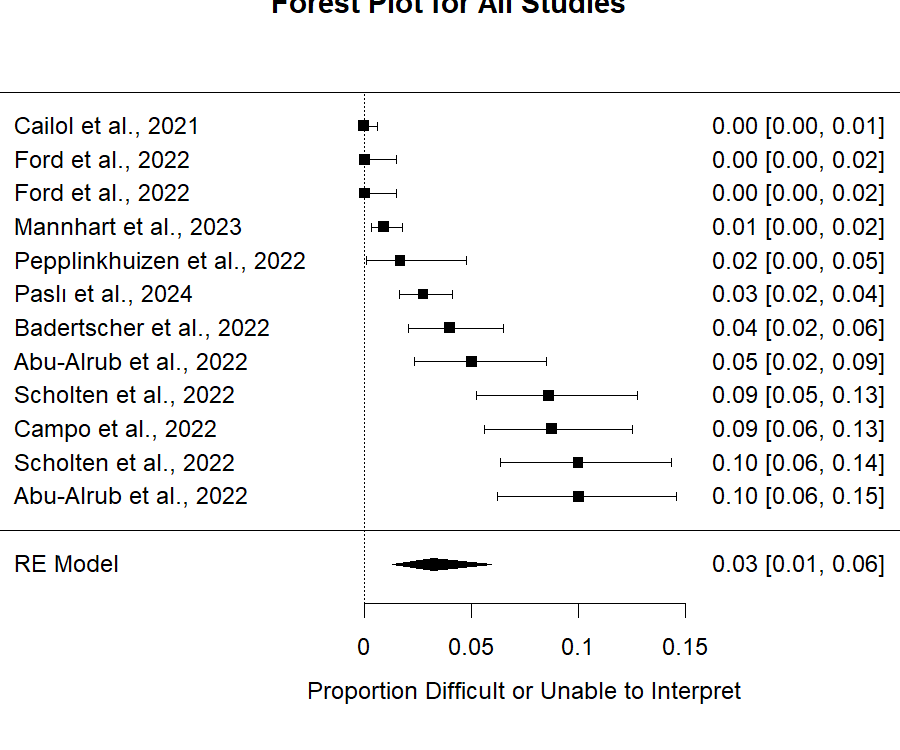


Figure S11. Forest plot for non-readable smartwatch ECGs excluding

Abu-Alrub et al., 2022 (outlier).

| 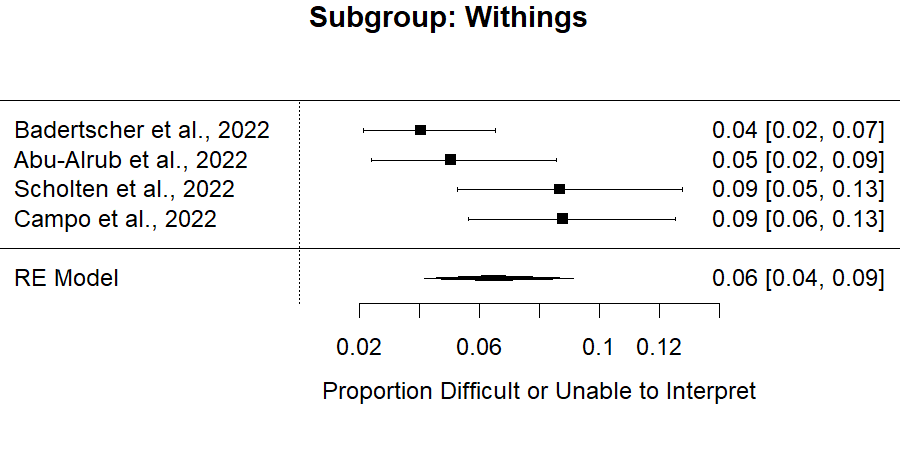 | 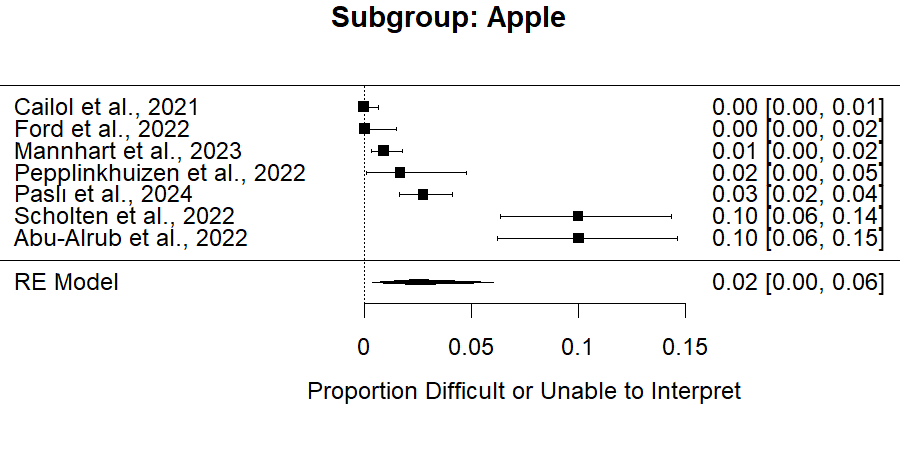 |
| --- | --- |
| (a) | (b) |

Figure S12. Forest plots for non-readable smartwatch ECGs from Apple Watch (a) and Withing Scanwatch (b).


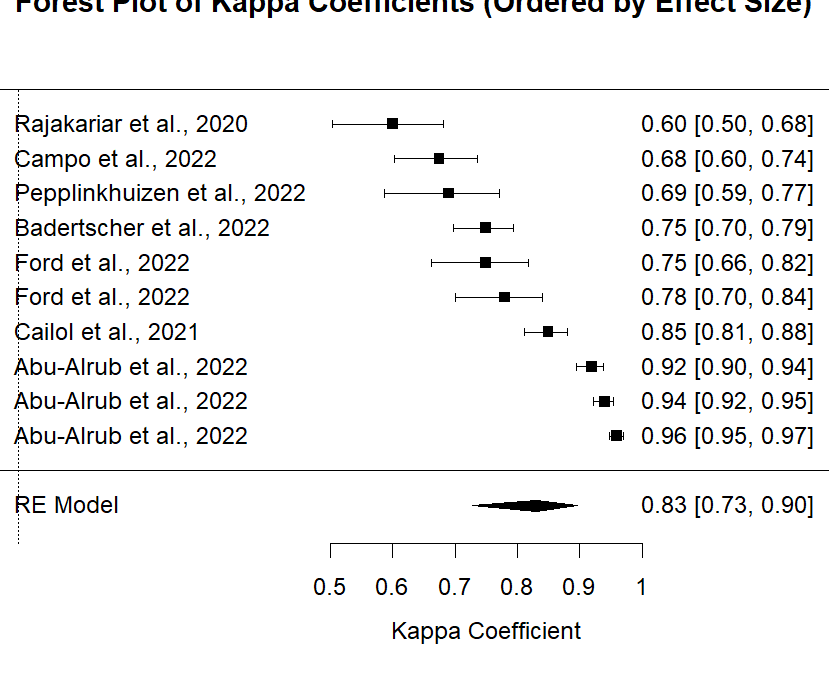


(a)


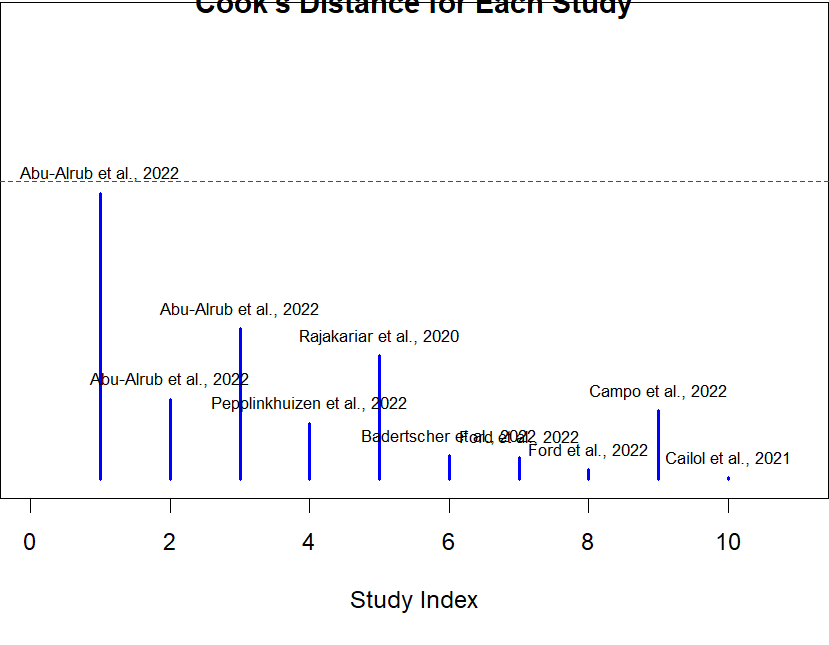


(b)

Figure S13. Forest plot (a) and Cook’s distance plot (b) for kappa interrater agreement on manual smartwatch ECG reading in distinguishing AFib and non-AFib.

|  |  |
| --- | --- |
| (a) | (b) |

Figure S14. Deek’s funnel plot for the ECG smartwatch diagnostic performance based on algorithmic (a) and manual readings (b).

| 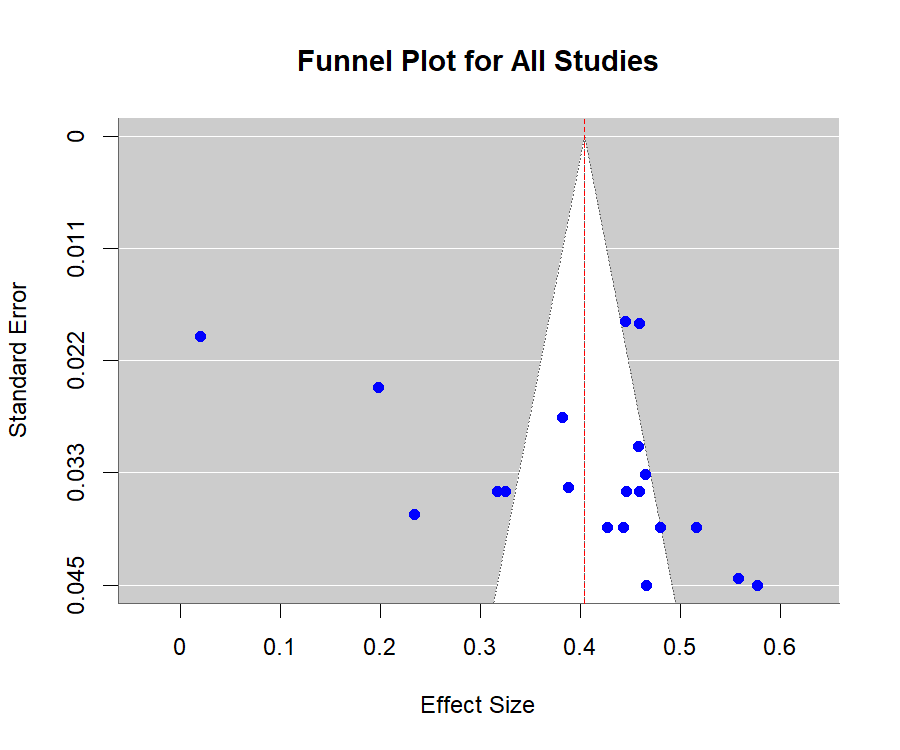  (a) *p*-Egg=0.014 | 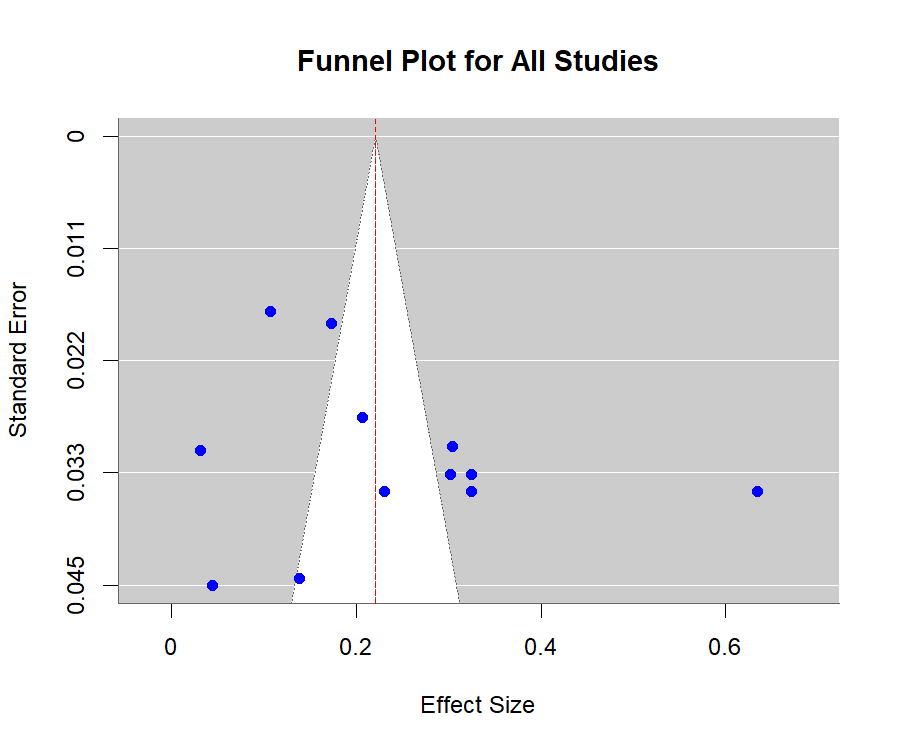  (b) *p*-Egg= 0.880 |
| --- | --- |
| 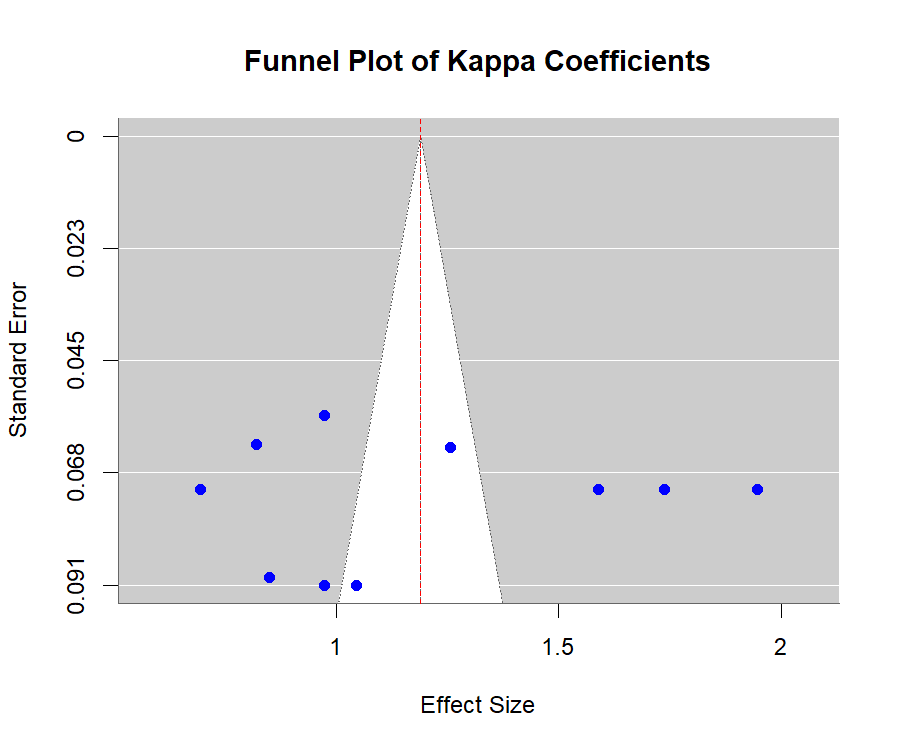  (c) *p*= 0.686 | |

Figure S15. Funnel plots for the uncertain result proportions based on algorithmic (a) and manual readings (b). Funnel plot for the interrater agrement coefficient kappa (c).
