# Supplementary material for: Accuracy and interpretability of smartwatch electrocardiogram for early detection of atrial fibrillation: A systematic review and meta‐analysis
Source: J Arrhythm. 2025 May 22;41(3):e70087. doi: 10.1002/joa3.70087 (PMC12096014; doi:10.1002/joa3.70087)
Supplement: Supplementary file 3 — Data S3. [file JOA3-41-e70087-s003.docx]

**Table S1.** Search strategies developed for PubMed database

| Search | Combination of keywords | Hits |
| --- | --- | --- |
| #1 | "smartwatch"[All Fields] OR "smartwatches"[All Fields] OR "portability"[All Fields] OR "portable"[All Fields] OR "portables"[All Fields] OR "single lead"[All Fields] | 54,357 |
| #2 | "electrocardiogram s"[All Fields] OR "electrocardiography"[MeSH Terms] OR "electrocardiography"[All Fields] OR "electrocardiogram"[All Fields] OR "electrocardiograms"[All Fields] OR "electrocardiography"[MeSH Terms] OR "electrocardiography"[All Fields] OR "ecg"[All Fields] | 272,148 |
| #3 | "atrial fibrillation"[All Fields] OR ("arrhythmia s"[All Fields] OR "arrhythmias, cardiac"[MeSH Terms] OR ("arrhythmias"[All Fields] AND "cardiac"[All Fields]) OR "cardiac arrhythmias"[All Fields] OR "arrhythmia"[All Fields] OR "arrhythmias"[All Fields]) OR ("tachycardia"[MeSH Terms] OR "tachycardia"[All Fields] OR "tachycardias"[All Fields] OR "tachycardia s"[All Fields]) | 351,220 |
| #4 | #1 AND #2 AND #3  ("atrial fibrillation"[All Fields] OR ("arrhythmia s"[All Fields] OR "arrhythmias, cardiac"[MeSH Terms] OR ("arrhythmias"[All Fields] AND "cardiac"[All Fields]) OR "cardiac arrhythmias"[All Fields] OR "arrhythmia"[All Fields] OR "arrhythmias"[All Fields]) OR ("tachycardia"[MeSH Terms] OR "tachycardia"[All Fields] OR "tachycardias"[All Fields] OR "tachycardia s"[All Fields])) AND ("electrocardiogram s"[All Fields] OR "electrocardiography"[MeSH Terms] OR "electrocardiography"[All Fields] OR "electrocardiogram"[All Fields] OR "electrocardiograms"[All Fields] OR ("electrocardiography"[MeSH Terms] OR "electrocardiography"[All Fields] OR "ecg"[All Fields])) AND ("smartwatch"[All Fields] OR "smartwatches"[All Fields] OR ("portability"[All Fields] OR "portable"[All Fields] OR "portables"[All Fields]) OR "single lead"[All Fields]) | 971 |

**Table S2.** List of excluded studies identified through databases or registries

| **Reason to exclude** | **Author** | **Year** | **Title** |
| --- | --- | --- | --- |
| No smart watch | Hyun et al. | 2024 | Evaluation of the Diagnostic Performance and Efficacy of Wearable Electrocardiogram Monitoring for Arrhythmia Detection after Cardiac Surgery |
| Review | Sinner et al. | 2024 | Screening for atrial fibrillation - what to do if the patient presents with atrial fibrillation on a smartwatch ECG? |
| Only citation | Li et al. | 2024 | Clinical value of continuous photoplethysmography algorithms for detection of atrial fibrillation by wearable devices |
| Not smartwatch | Savickas et al. | 2024 | Screening for atrial fibrillation in care homes using pulse palpation and the AliveCor Kardia MobileÂ® device: a comparative cross-sectional pilot study |
| Not smartwatch | Gruwez et al. | 2024 | Real-world validation of smartphone-based photoplethysmography for rate and rhythm monitoring in atrial fibrillation |
| Not Afib | Grautoff and Watol | 2024 | Life-saving fall detection by aÂ smartwatch in aÂ case of ventricular fibrillation |
| Not Afib (combined with atrial flutter) | Bumgarner et al. | 2018 | Smartwatch algorithm for automated detection of atrial fibrillation |
| 12 lead is not mentioned | Perez et al. | 2019 | Large-scale assessment of a smartwatch to identify atrial fibrillation |
| 12 lead is not mentioned | Avram et al. | 2021 | Validation of an algorithm for continuous monitoring of atrial fibrillation using a consumer smartwatch |
| Not ECG | Tison et al. | 2018 | Passive detection of atrial fibrillation using a commercially available smartwatch |
| Not suitable study design | Strik et al. | 2021 | Smartwatch-based detection of cardiac arrhythmias: Beyond the differentiation between sinus rhythm and atrial fibrillation |
| Not ECG | Dörr et al. | 2019 | The WATCH AF trial: SmartWATCHes for detection of atrial fibrillation |
| 12 lead is not mentioned | Wasserlauf et al. | 2019 | Smartwatch performance for the detection and quantification of atrial fibrillation |
| Not Afib | Strik et al. | 2023 | The use of smartwatch electrocardiogram beyond arrhythmia detection |
| No outcome of interest | Shih et al. | 2022 | Direct-to-consumer detection of atrial fibrillation in a smartwatch electrocardiogram: medical overuse, medicalisation and the experience of consumers |
| 12 lead is not mentioned | Chang et al. | 2022 | Atrial fibrillation detection using ambulatory smartwatch photoplethysmography and validation with simultaneous holter recording |
| Review | Tajrishi et al. | 2019 | Smartwatch for the detection of atrial fibrillation |
| Wrong document type | Benezet-Mazuecos et al. | 2018 | Smart devices for a smart detection of atrial fibrillation |
| No outcome of interest | Inui et al. | 2020 | Use of a smart watch for early detection of paroxysmal atrial fibrillation: validation study |
| 12 lead is not mentioned | Krivoshei et al. | 2017 | Smart detection of atrial fibrillation |
| only abstract | Scholten et al. | 2021 | A comparison of over-the-counter available smartwatches and devices for electrocardiogram based detection of atrial fibrillation |
| Not ECG | Bashar et al. | 2019 | Smartwatch based atrial fibrillation detection from photoplethysmography signals |
| Not ECG | Bashar et al. | 2019 | Atrial fibrillation detection from wrist photoplethysmography signals using smartwatches |
| Wrong document type | Scquizzato et al. | 2021 | Smartwatch-detected atrial fibrillation in the Emergency Department: possible implications and treatment |
| 12 lead is not mentioned | Dobrev et al. | 2020 | Smart device-based detection of atrial fibrillation: Opportunities and challenges in the emerging world of digital health |
| Not suitable study design | Koshy et al. | 2018 | Smart watches for heart rate assessment in atrial arrhythmias |
| Sigle lead | Zimerman et al. | 2020 | Trends in New Diagnoses of Atrial Fibrillation After Release of an ECG-Capable Smartwatch |
| 12 lead is not mentioned | Ding et al. | 2022 | Usability of a smartwatch for atrial fibrillation detection in older adults after stroke |
| Not suitable study design | Kalla et al. | 2019 | SMART about watches: we need technical and biological validation of atrial fibrillation screening |
| only abstract | Rajakariar et al. | 2019 | P572 Accuracy of a smartwatch based single-lead electrocardiogram device in screening for atrial fibrillation |
| Not ECG | Liao et al. | 2022 | Impact of recording length and other arrhythmias on atrial fibrillation detection from wrist photoplethysmogram using smartwatches |
| only abstract | Fiorina et al. | 2022 | Smartwatch-based detection of atrial arrhythmia using a deep neural network in a tertiary care hospital |
| No outcome of interest | Nasarre et al. | 2022 | Using a smartwatch electrocardiogram to detect abnormalities associated with sudden cardiac arrest in young adults |
| 12 lead is not mentioned | Hermans et al. | 2023 | Accuracy of continuous photoplethysmography-based 1 min mean heart rate assessment during atrial fibrillation |
| Not ECG | Nemati et al. | 2016 | Monitoring and detecting atrial fibrillation using wearable technology |
| Wrong document type | Burri et al. | 2018 | Screening for atrial fibrillation using smartphones and smartwatches |
| only abstract | Otto | 2020 | Heartbeat: smartwatch devices for detection of atrial fibrillation |
| No outcome of interest | Koh et al. | 2021 | Smartphone electrocardiogram for detecting atrial fibrillation after a cerebral ischaemic event: a multicentre randomized controlled trial |
| Not suitable study design | Duncker et al. | 2021 | Smart wearables for cardiac monitoringâ€”real-world use beyond atrial fibrillation |
| Not ECG | Zhao et al. | 2024 | Evaluation of an algorithmâ€guided photoplethysmography for atrial fibrillation burden using a smartwatch |
| Not ECG | Mutke et al. | 2021 | Comparison and combination of single-lead ECG and photoplethysmography algorithms for wearable-based atrial fibrillation screening |
| Not suitable study design | Guo et al. | 2019 | Mobile photoplethysmographic technology to detect atrial fibrillation |
| Not suitable study design | Chon et al. | 2018 | Detection of atrial fibrillation using a smartwatch |
| Not smartwatch | Saarinen et al. | 2023 | Wrist-worn device combining PPG and ECG can be reliably used for atrial fibrillation detection in an outpatient setting |
| Sigle lead | Lubitz et al. | 2022 | Detection of atrial fibrillation in a large population using wearable devices: the Fitbit Heart Study |
| 12 lead is not mentioned | Reissenberger et al. | 2023 | Determine atrial fibrillation burden with a photoplethysmographic mobile sensor: the atrial fibrillation burden trial: detection and quantification of episodes of atrial fibrillation using a cloud analytics service connected to a wearable with photoplethysmographic sensor |
| 12 lead is not mentioned | Wasserlauf et al. | 2023 | Accuracy of the Apple watch for detection of AF: A multicenter experience |
| No outcome of interest | Al-Kaisey et al. | 2020 | Accuracy of wrist-worn heart rate monitors for rate control assessment in atrial fibrillation |
| only abstract | Gruwez et al. | 2021 | Performance of an artificial intelligence algorithm to detect atrial fibrillation on a 24-hour continuous photoplethysmography recording using a smartwatch: ACURATE study |
| only abstract | Ding et al. | 2021 | Use of a smartwatch and app designed by stroke survivors for atrial fibrillation detection in older adults after stroke/transient ischemic event: Preliminary findings from an ongoing randomized clinical trial |
| only abstract | Briosa et al. | 2023 | Automated atrial fibrillation detection with a smartwatch and smart-ring in individuals with cardiovascular disease |
| 12 lead is not mentioned | Han et al. | 2023 | A smartwatch system for continuous monitoring of atrial fibrillation in older adults after stroke or transient ischemic attack: application design study |
| no smart watch | Yao et al. | 2023 | Factors affecting wearable ECG device adoption by general practitioners for atrial fibrillation screening: cross-sectional study |
| 12 lead is not mentioned | Ding et al. | 2023 | Accuracy, Usability, and Adherence of Smartwatches for Atrial Fibrillation Detection in Older Adults After Stroke: Randomized Controlled Trial |
| no smart watch | Wegner et al. | 2020 | Prospective blinded Evaluation of the smartphone-based AliveCor Kardia ECG monitor for Atrial Fibrillation detection: The PEAK-AF study |
| Sigle lead | Ding, Eric Y | 2021 | Feasibility of smartwatch-based atrial fibrillation detection among older adults after stroke |
| 6 lead | Bacevicius et al. | 2022 | High specificity wearable device with photoplethysmography and six-lead electrocardiography for atrial fibrillation detection challenged by frequent premature contractions: doubleCheck-AF |
| only abstract | Racine et al. | 2022 | PO-631-04 ACCURACY OF A SMARTWATCH ECG TO DIAGNOSE ATRIAL FIBRILLATION AND NORMAL SINUS RHYTHM |
| Not ECG | Nonoguchi et al. | 2022 | Accuracy of wristwatch-type photoplethysmography in detecting atrial fibrillation in daily life |
| No outcome of interest | Perino et al. | 2021 | Arrhythmias Other Than Atrial Fibrillation in Those With an Irregular Pulse Detected With a Smartwatch |
| only abstract | Lee et al. | 2023 | The performance of preliminary version of irregular heart rhythm notification algorithm of a consumer smartwatch for screening of atrial fibrillation |
| 12 lead is not mentioned | Mohagheghian et al. | 2024 | Atrial fibrillation detection on reconstructed photoplethysmography signals collected from a smartwatch using a denoising autoencoder |
| Case report | Bedi et al. | 2023 | The integration of artificial intelligence into patient care: a case of Atrial Fibrillation Caught by a Smartwatch |
| No outcome of interest | Zhang et al. | 2021 | Better Battery Life: Towards Energy-Efficient Smartwatch-Based Atrial Fibrillation Detection in Ambulatory Free-living Environments |
| only abstract | Mannhart et al. | 2022 | Clinical validation of five direct-to-consumer smartwatches to detect atrial fibrillation in a real-world cohort of patients |
| No outcome of interest | Marcus | 2022 | Smartwatch-Detected Atrial Fibrillation: The "value" in the Positive Predictive Value |
| only abstract | Pepplinkhuizen et al. | 2021 | Accuracy of the Apple Watch Electrocardiogram to Detect Atrial Fibrillation in a Real-World Clinical Setting |
| Not smartwatch | Rizas et al. | 2022 | Smartphone-based screening for atrial fibrillation: a pragmatic randomized clinical trial |
| Not ECG | Meza Burgos et al. | 2023 | Accuracy of a Smartwatch to Assess Heart Rate Monitoring and Atrial Fibrillation in Stroke Patients |
| Wrong document type | Giancaterino and Hsu | 2018 | The Smartwatch Will See You Now: Implications of Mass Screening for Atrial Fibrillation |
| only abstract | Wasserlauf et al. | 2018 | Accurate Detection and Quantification of Atrial Fibrillation Using a Smartwatch with ECG Watchband. |
| No outcome of interest | Fabritz et al. | 2022 | Smartphone and wearable detected atrial arrhythmias in Older Adults: Results of a fully digital European Case finding study |
| not english | Haverkamp et al. | 2022 | [Diagnosing atrial fibrillation using a smartwatch]. |
| No outcome of interest | Teh and DÃrr | 2023 | Smart Devices in Detecting AF: Excellent Signal Quality, But AI Can Still Learn from Clinicians. |
| only abstract | Vandenberk et al. | 2018 | Atrial Fibrillation Diagnosis Based on a Smartphone Derived Ppg Waveform: A Diagnostic Accuracy Study versus Single-Lead |
| only abstract | e Gala et al. | 2023 | PO-04-179 Diagnostic Accuracy of The Apple Watch and Cart-I Ring for Detecting Atrial Arrhythmias (AF, AT, and Atrial Flutters) |

**Table S3.** List of excluded studies identified through additional method

| Reason to exclude | Author | Year | Title |
| --- | --- | --- | --- |
| No outcome of interest | Seshadri et al. | 2019 | Accuracy of the Apple Watch 4 to measure heart rate in patients with atrial fibrillation |
| No outcome of interest | Hwang et al. | 2019 | Assessing accuracy of wrist-worn wearable devices in measurement of paroxysmal supraventricular tachycardia heart rate |
| Not Afib | Sequeira et al. | 2020 | Common wearable devices demonstrate variable accuracy in measuring heart rate during supraventricular tachycardia |
| No outcome of interest | Leroux et al. | 2023 | Feasibility and diagnostic value of recording Smartwatch electrocardiograms in neonates and children |
| Not ECG | Koshy et al. | 2018 | Smart watches for heart rate assessment in atrial arrhythmias |
| Not ECG | Han et al. | 2020 | Digital image processing features of smartwatch photoplethysmography for cardiac arrhythmia detection |
| Not compared with 12-lead ECG | Wasserlauf et al. | 2023 | Accuracy of the Apple watch for detection of AF: A multicenter experience |
| Not compared with 12-lead ECG | Chang et al. | 2022 | Atrial fibrillation detection using ambulatory smartwatch photoplethysmography and validation with simultaneous holter recording |
| No outcome of interest | Wyatt et al. | 2020 | Clinical evaluation and diagnostic yield following evaluation of abnormal pulse detected using Apple Watch |
| Prediction of heart rythm sinus versus AFib | Lee et al. | 2022 | Comparison of Apple watch vs KardiaMobile: A tale of two devices |
| Not suitable study design | Roelle | 2022 | Expanding telehealth through technology: Use of digital health technologies during pediatric electrophysiology telehealth visits |
| Not ECG | Liao | 2022 | Impact of recording length and other arrhythmias on atrial fibrillation detection from wrist photoplethysmogram using smartwatches |
| Not ECG | Dörr | 2019 | The WATCH AF trial: SmartWATCHes for detection of atrial fibrillation |
| No outcome of interest | Liu | 2022 | Wearable smartwatch facilitated remote health management for patients undergoing transcatheter aortic valve replacement |
| No outcome of interest | Feldman | 2022 | Will Apple devices' passive atrial fibrillation detection prevent strokes? Estimating the proportion of high-risk actionable patients with real-world user data |
| Not Afib (combined with atrial flutter) | Bumgarner | 2018 | Smartwatch algorithm for automated detection of atrial fibrillation |
| Not smartwatch | Desteghe | 2017 | Performance of handheld electrocardiogram devices to detect atrial fibrillation in a cardiology and geriatric ward setting |
| No outcome of interest | Perez | 2019 | Large-scale assessment of a smartwatch to identify atrial fibrillation |
| Not ECG | Bashar | 2019 | Atrial fibrillation detection from wrist photoplethysmography signals using smartwatches |
| Not ECG | Guo | 2019 | Mobile photoplethysmographic technology to detect atrial fibrillation |
| Not ECG | Zhang | 2019 | Validation of single centre pre-mobile atrial fibrillation apps for continuous monitoring of atrial fibrillation in a real-world setting: pilot cohort study |
| Not ECG - PPG in Apple Watch early series | Tison | 2018 | Passive detection of atrial fibrillation using a commercially available smartwatch |
| Not smartwatch | Halcox | 2017 | Assessment of Remote Heart Rhythm Sampling Using the AliveCor Heart Monitor to Screen for Atrial Fibrillation: The REHEARSE-AF Study |
| Not smartwatch/Wrist-worn device | Saarinen | 2023 | Wrist-worn device combining PPG and ECG can be reliably used for atrial fibrillation detection in an outpatient setting |
| Not smartwatch | Yuniadi | 2024 | Prevalence of atrial fibrillation based on tertiary hospital survey in Indonesia: A smartphone-based diagnosis |
| Not Afib (combined with atrial flutter) | Yalin | 2023 | Diagnostic accuracy of Apple Watch Series 6 recorded single-lead ECGs for identifying supraventricular tachyarrhythmias: a comparative analysis with invasive electrophysiological study |
| Not Afib | Ernstsson | 2024 | Validation of smartwatch electrocardiogram intervals in children compared to standard 12 lead electrocardiograms |
| Not smartwatch | Turnbull | 2024 | Accuracy of a Single-Lead ECG Device for Diagnosis of Cardiac Arrhythmias Compared Against Cardiac Electrophysiology Study |
| Not ECG | Meza | 2024 | Accuracy of a Smartwatch to Assess Heart Rate Monitoring and Atrial Fibrillation in Stroke Patients |
| Not ECG | Selder | 2024 | Accuracy of a Standalone Atrial Fibrillation Detection Algorithm Added to a Popular Wristband and Smartwatch: Prospective Diagnostic Accuracy Study |
| No outcome of interest | Sprenger | 2022 | Feasibility and Reliability of Smartwatch to Obtain Precordial Lead Electrocardiogram Recordings |
| Not Afib | Paech | 2021 | Accuracy of the Apple Watch single-lead ECG recordings in pre-term neonates |
| Not Afib | Kobel | 2022 | Accuracy of the Apple Watch iECG in Children With and Without Congenital Heart Disease |
| No outcome of interest | de Zande | 2023 | Using a Smartwatch to Record Precordial Electrocardiograms: A Validation Study |
| Not Afib | Nasarre | 2022 | Using a smartwatch electrocardiogram to detect abnormalities associated with sudden cardiac arrest in young adults |
| Not suitable study design | Leroux | 2022 | Using a smartwatch to record an electrocardiogram in the pediatric population |
| Not ECG | Ibrahim | 2024 | Evaluation of Wrist‑Worn Photoplethysmography Trackers with an Electrocardiogram in Patients with Ischemic Heart Disease: A Validation Study |
| Not smartwatch | Fu and Li | 2021 | Diagnostic performance of a wearing dynamic ECG recorder for atrial fibrillation screening: the HUAMI heart study |

Table S4. Sensitivity and specificity of smartwatch ECG in detecting AFib compared to 12-lead ECG

| Author, Year [Ref] | Brand | Automatic Detection | | Manual Detection | | |
| --- | --- | --- | --- | --- | --- | --- |
|  |  | Unclassified | Qualitative | Hard to or unable to read | Kappa | Qualitative |
| Ploux et al., 2022 [28] | Apple Watch Series 4 | NA | NA | NR |  |  |
| Abu-Alrub et al., 2022 [10] | Apple Watch Series 5 | 19 | Poor recording, HR<50 bpm, HR>150 bpm | 20 | 0.96 | Excessive artifact or baseline wander |
|  | Samsung Galaxy Watch Active 3 | 20 | Poor recording, HR<50 bpm | 70 | 0.92 | Excessive artifact or baseline wander |
|  | Withings Move ECG | 37 | Poor recording, HR<50 bpm, HR>100 bpm | 10 | 0.94 | Excessive artifact or baseline wander |
| Mannhart et al., 2023 [29] | Apple Watch Series 6 | 30 | HR<50 bpm, HR>150 bpm, poor recording | 9 of 825 | NR | Low quality |
|  | Samsung Galaxy Watch 3 | 28 | HR<50 bpm, HR>120 bpm, poor recording |  |  |  |
|  | Withings Scanwatch | 40 | Low/high HR, poor recording |  |  |  |
|  | Fitbit Sense | 35 | HR<50 bpm, HR>120 bpm |  |  |  |
| Racine et al., 2022 [11] | Apple Watch Series 5 | 136 | HR<50 bpm, HR>120 bpm, poor recording | NR | NR | NR |
| Pengel et al., 2023 [30] | Withings Scanwatch | 9 | Noise, QRS-affecting artifacts | NA | NA | NA |
| Pepplinkhuizen et al., 2022 [27] | Apple Watch Series 6 | 36 | HR< 50 bpm, poor recording | 2 | 0.69 |  |
| Rajakariar et al., 2020 [13] | AliveCor KardiaBand (attached to Apple Watch) | 39 | Low/high HR, artifact, other cardiac anomalies (i.e. premature atrial complexes, first degree AV block) | NR | 0.60 | Excessive artifact or baseline wander |
| Badertscher et al., 2022 [16] | Withings Scanwatch | 44 | Low/high HR, motion artifact | 13 | 0.75 | NR |
| Ford et al., 2022 [31] | Apple Watch Series 4 | 37 | Low/high HR, poor recording | 0 | 0.75 | Excessive artifact or baseline wander |
|  | AliveCor KardiaBand | 25 | Low/high HR, poor recording | 0 | 0.78 | Excessive artifact or baseline wander |
| Campo et al., 2022 [32] | Withings Scanwatch | 51 | Noise | 23 | 0.68 | NR |
| Chen et al., 2020 [33] | Amazfit Health Band | 15 | Noise | NR | NA | NR |
| Cunha et al., 2020 [25] | AliveCor KardiaBand | 29 | Poor quality | NA | NA | NR |
| Müller et al., 2024 [26] | Apple Watch Series 5 | NA | NA | NA | NA | NR |
| Paslı et al., 2024 | Apple Watch Series 7 | NA | NA | 21 | NR | Inconclusive recording due to artifacts and noise |
| Caillol et al., 2021 [9] | Apple Watch Series 4 and 5 | NA | NA | 0 | 0.85 | Baseline wanders and motion artifacts are common |
| Velraeds et al., 2023 [35] | Apple Watch Series 5 | 142 | Inconclusive recording | NA | NA | NA |
| Scholten et al., 2022 [36] | Apple Watch Series 5 | 44 | Inconclusive, high/low HR, unspecified | 22 | NR | NR |
|  | Withings Scanwatch | 44 | Inconclusive, high/low HR | 19 | NR | NR |
| Niu et al., 2023 [37] | Huawei Watch GT2 Pro | 0 | NA | NA | NA | NA |

NR, not reported; Not applicable
